# Supplementary material for: Biologically informed NeuralODEs for genome-wide regulatory dynamics
Source: Genome Biol. 2024 May 21;25:127. doi: 10.1186/s13059-024-03264-0 (PMC11106922; doi:10.1186/s13059-024-03264-0)
Supplement: Supplementary file 1 — Additional file 1: Supplemental results. This file contains Supplementary Figures S1-S12 and Tables S1-S16. [file 13059_2024_3264_MOESM1_ESM.pdf]

Supplemental results accompanying: "Biologically informed neural ordinary differential equations for genome-wide regulatory dynamics"

Intekhab Hossain[1*], Viola Fanfani[1], Jonas Fischer[1], John Quackenbush[1] and Rebekka Burkholz[2]

[1*]Department of Biostatistics, Harvard T.H. Chan School of Public Health, Boston, MA, USA.
[2]Helmholtz Center for Information Security (CISPA), Saarbrücken, Germany.

*Corresponding author(s). E-mail(s): ihossain@g.harvard.edu;
Contributing authors: vfanfani@hsph.harvard.edu;
jfischer@hsph.harvard.edu; johnq@hsph.harvard.edu;
burkholz@cispa.de;

This is the supplemental results file accompanying the paper. For further queries, please feel free to reach out to `ihossain@g.harvard.edu`

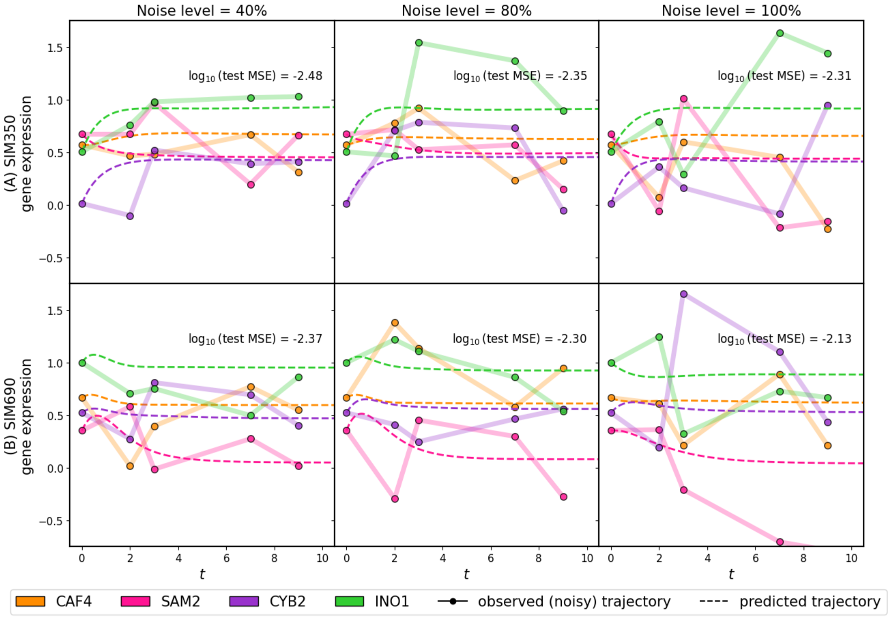

Additional File 1: Figure S1 We applied PHOENIX to simulated gene expression data originating from two different *in silico* dynamical systems SIM350 (A) and SIM690 (B) that simulate the temporal expression of 350 and 690 genes respectively. Each simulated trajectory consisted of five time points ($t = 0, 2, 3, 7, 9$) and was subjected to varying levels of Gaussian noise ($\frac{\text{noise } \sigma}{\text{mean}} = 40\%, 80\%, 100\%$). **These results are provided in addition to the noise settings of 0%, 5%, 10%, and 20% that have been shown in Figure 2 of the main paper**. Since PHOENIX uses a user-defined prior network model as a regularizer, we also corrupted the prior models up to an amount commensurate with the noise level. For each noise setting we trained PHOENIX on 140 of these "observed" trajectories and validated on 10. The performance on the validation trajectories was used to determine the optimal value of λ_{prior}. We then tested the trained model (with the optimal choice of λ_{prior}) on 10 new test set trajectories. We display both observed and predicted test set trajectories for four arbitrary genes in both SIM350 and SIM690, across all noise settings. We display the mean squared error (MSE) between the predictions and the 10 pre-noise test set trajectories.

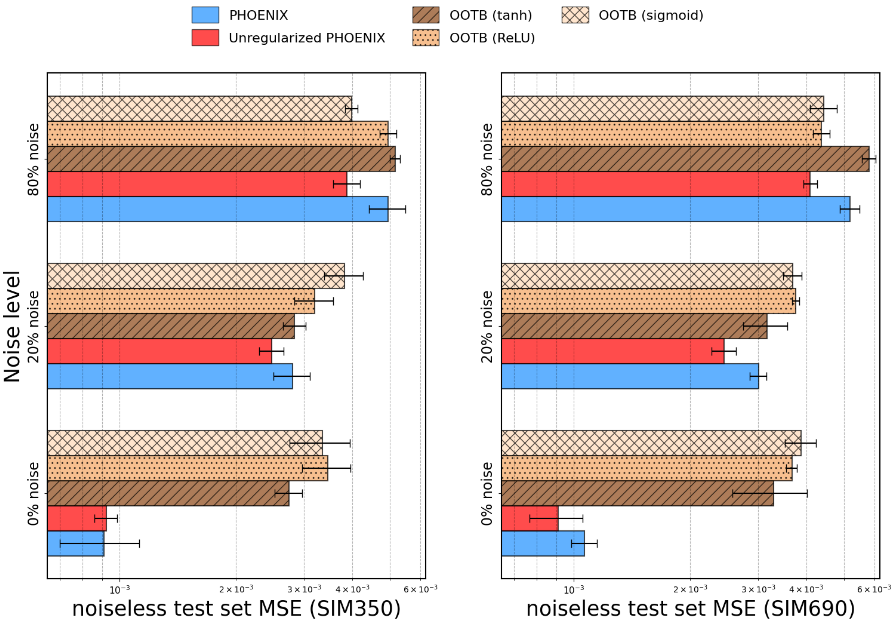

Additional File 1: Figure S2 We measured the marginal contribution of PHOENIX's architecture and incorporation of prior information by comparing against the baseline contributions of out-of-the-box NeuralODE models with three different activation functions, across both *in silico* dynamical systems SIM350 and SIM690, after subjecting the data to different amounts of noise. We display the performance of the out-of-the-box models, as well as PHOENIX (λ_{prior} tuned using the validation set) and its unregularized version ($\lambda_{\text{prior}} = 0$), in terms of how well held out time points from noiseless test set trajectories could be predicted after training on trajectories from different noise settings. The entire procedure was repeated five times to generate average mean-squared error (MSE) values and error bars.

Additional File 1: Table S1 Assessing the contribution of priors to PHOENIX explainability, as well as the effect of prior misspecification, in both *in silico* experiments and real experimental data

	Genes	Noise	PHOENIX	Prior constraints
SIM350[1]	350	0%	0.987	1.000
	350	5%	0.980	0.970
	350	10%	0.979	0.950
	350	20%	0.943	0.900
	350	40%	0.761	0.800
	350	80%	0.538	0.600
	350	100%	0.520	0.500
SIM690[1]	690	0%	0.986	1.000
	690	5%	0.984	0.975
	690	10%	0.983	0.950
	690	20%	0.958	0.900
	690	40%	0.737	0.800
	690	80%	0.531	0.600
	690	100%	0.529	0.500
Yeast[2]	3551	-	0.934	0.790
Breast[2]	500	-	0.958	0.820
	2000	-	0.906	0.840
	4000	-	0.909	0.810
	11165	-	0.904	0.810

[1]For *in silico* experiments (SIM350, SIM690) the prior knowledge model was corrupted by an amount commensurate with the noise level (see Additional File 2: Section 4.2). Then for each scenario, a network representation of the misspecified prior model was checked for how well it aligned with the ground truth GRN in terms of AUC. This AUC was compared to that obtained by aligning the ground truth GRN against a network extracted from a PHOENIX model trained using the misspecified prior in question.

[2]This same approach of aligning a "ground truth" GRN against extracted and prior networks was repeated for the real data sets (yeast cell cycle and breast cancer). But this time the prior networks came from the motiff-prior models used in model-fitting (see Methods 2.2 and 3.2), and the "ground-truth" GRNs were experimentally verified ChIP-Seq networks describing transcription factor binding.

Additional File 1: Table S2 Assessing the effect on explainability of withholding complete subsets of information from the prior ($\mathcal{P}^*$), in *in silico* experiments. Gaussain noise of 5% was added to all expression values.

	% of edges withheld[1] from prior model $\mathcal{P}^*$	[2]AUC when discriminating between non-existent edges vs.		
		all true edges	true edges in $\mathcal{P}^*$	true edges withheld from $\mathcal{P}^*$
SIM350	0%	0.98	0.98	N/A
	5%	0.97	0.97	0.93
	10%	0.97	0.97	0.94
	25%	0.97	0.98	0.92
	50%	0.93	0.98	0.88
	75%	0.90	0.99	0.87
	100%	0.86	N/A	0.86
SIM690	0%	0.98	0.98	N/A
	5%	0.97	0.97	0.98
	10%	0.96	0.97	0.95
	25%	0.98	0.98	0.95
	50%	0.96	0.98	0.93
	75%	0.92	0.98	0.91
	100%	0.86	N/A	0.86

[1]For *in silico* experiments (SIM350, SIM690) under 5% noise, the prior knowledge model $\mathcal{P}^*$ was rendered "incomplete" by setting a certain percentage of nonzero prior interactions to 0 (see Additional File 2: Section 4.2).

[2]For each scenario, an AUC was calculated by aligning the entire ground truth GRN against a network extracted from a PHOENIX model trained using the incomplete prior in question. AUC values were also separately calculated for subsets of the ground truth GRN edges based on whether or not the edge was withheld from $\mathcal{P}^*$.

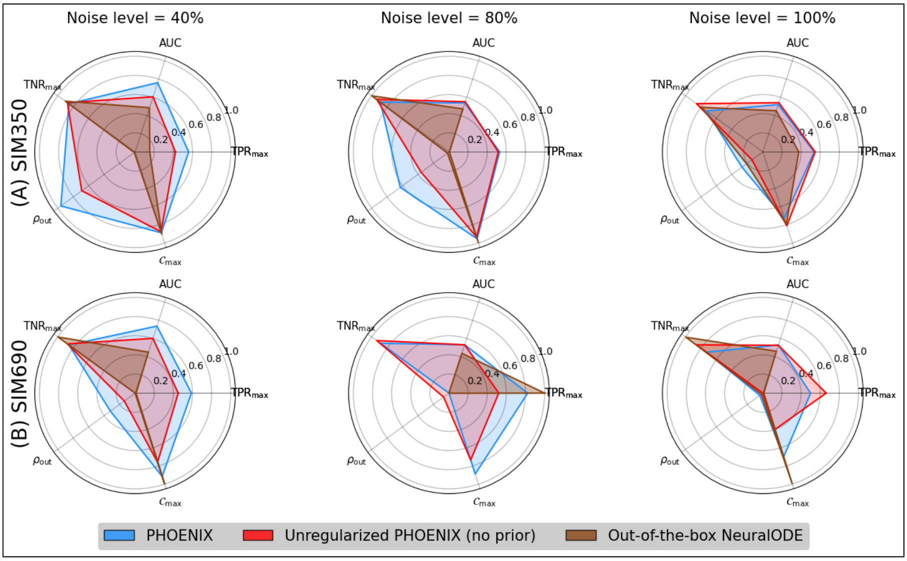

Additional File 1: Figure S3 We extracted encoded GRNs from the trained PHOENIX models and the best-performing out-of-the-box NeuralODE models, for both *in silico* dynamical systems SIM350 (A) and SIM690 (B) across all noise settings. Here we display noise settings 40%, 80% and 100%. **These results are provided in addition to the noise settings of 0%, 5%, 10%, and 20% that have been shown in Figure 3 of the main paper**.We compared these GRN estimates to the corresponding ground truth GRNs used to formulate SIM350 and SIM690, and obtained AUC values as well as out-degree correlations (ρ_{out}). We also reverse-engineered a metric ($\mathcal{C}_{\max}$) to inform how sparsely PHOENIX had inferred the dynamics (see Additional File 2: Section 2). Furthermore, we used these $\mathcal{C}_{\max}$ values to obtain optimal true positive and true negative rates ($\text{TPR}_{\max}$ and $\text{TNR}_{\max}$) that were independent of any cutoff value, allowing us to compare between "best possible" networks across all settings.

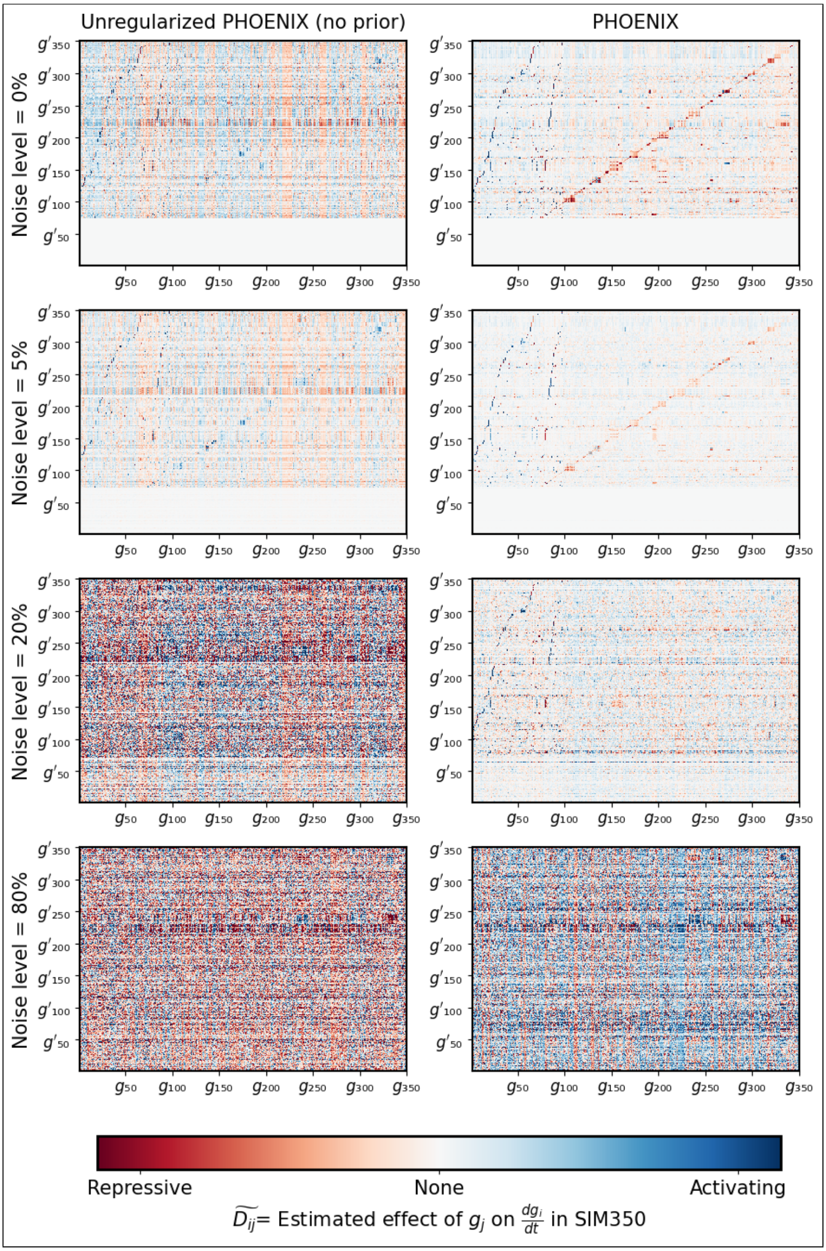

Additional File 1: Figure S4 We investigated *how* PHOENIX used prior information to sparsify the learned dynamics by computing the encoded 350×350 dynamics matrices $\widetilde{\boldsymbol{D}}$ (see Additional File 2: Section 2), across multiple noise settings in SIM350. We display $\widetilde{\boldsymbol{D}}$ for both PHOENIX as well as its unregularized ($\lambda_{\text{prior}} = 0$) version as heatmaps of effect size. Unlike its unregularized counterpart, PHOENIX identifies core elements of the dynamics even at relatively high noise levels.

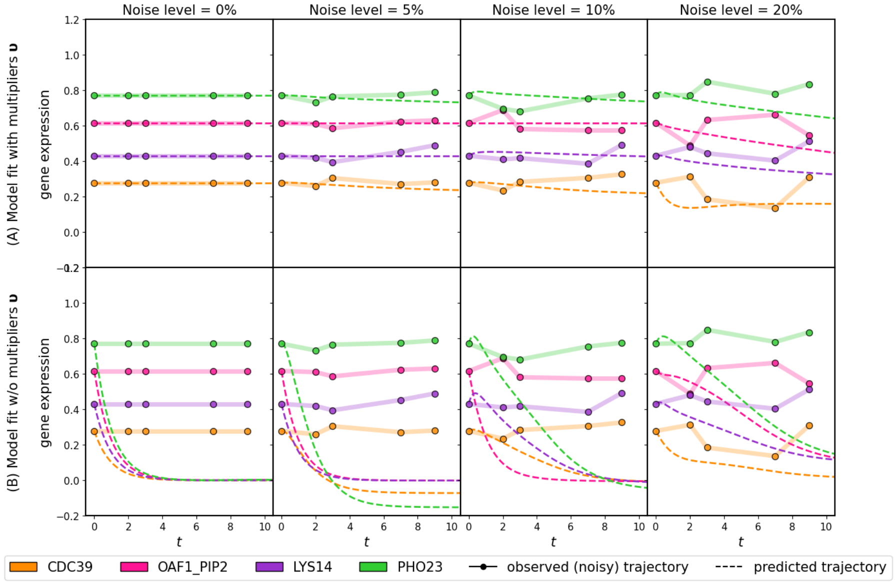

Additional File 1: Figure S5 PHOENIX uses gene-specific multipliers $\boldsymbol{\upsilon} \in \mathbb{R}^n$ (see Model Formulation in main paper) to simplify the representation of steady state for genes without upstream transcription factors $\left(\frac{dg_i(t)}{dt} = 0, \forall t\right)$. In order to explore the contribution of these gene-specific multipliers towards the PHOENIX model, we tested PHOENIX with (A) and without (B) the multipliers using gene expression data originating from the *in silico* dynamical system SIM690. We used SIM690 to simulate the temporal gene expression patterns for its 690 genes. Each simulated trajectory consisted of five time points ($t = 0, 2, 3, 7, 9$) and was subjected to varying levels of Gaussian noise ($\frac{\text{noise } \sigma}{\text{mean}}$ = 0%, 5%, 10%, and 20%). We also corrupted the user-defined prior models up to an amount commensurate with the noise level. We display across all noise settings both observed and predicted trajectories for four arbitrary genes that had true flat trajectories i.e $\left(\frac{dg_i(t)}{dt} = 0, \forall t\right)$ in SIM690.

Additional File 1: Table S3 In order to incorporate prior domain knowledge, PHOENIX uses an adjacency matrix $\boldsymbol{A}$ of likely network structure based on user-defined biological insights (including experimentally validated interactions, motif map of promoter targets, etc.). Here, $\boldsymbol{A}_{ij} \in \{+1, -1, 0\}$ representing an activating, repressive, or no prior interaction, respectively. But for real world scenario, the signs (activating/repressive) of prior interactions are often unknown. So we wanted to check whether formulating $\boldsymbol{A}$ simply based on *prior interaction existence*, $\boldsymbol{A}_{ij} \in \{+1, 0\}$, would suffice. Below we show the results of testing the importance of providing the correct prior signs to PHOENIX using our *in silico* setup where the ground truth signs were always known. We tested multiple forms of regularized PHOENIX models and report results both in terms predictive accuracy (MSE on test set) and explainability (AUC from comparing inferred dynamics to ground truth GRNs).

		Predictive performance (MSE)			Explainability (AUC)		
	Noise	PHX_0[1]	PHX_+[2]	PHX[3]	PHX_0[1]	PHX_+[2]	PHX[3]
SIM350	0%	0.0009	0.0015	0.0009	0.94	0.97	0.99
	5%	0.0010	0.0012	0.0011	0.90	0.97	0.98
	10%	0.0019	0.0019	0.0014	0.86	0.94	0.98
	20%	0.0025	0.0029	0.0028	0.77	0.92	0.94
	40%	0.0031	0.0039	0.0037	0.61	0.75	0.76
	80%	0.0039	0.0049	0.0050	0.55	0.54	0.54
	100%	0.0045	0.0055	0.0059	0.54	0.53	0.52
SIM690	0%	0.0009	0.0012	0.0011	0.85	0.98	0.99
	5%	0.0018	0.0016	0.0012	0.80	0.97	0.98
	10%	0.0024	0.0021	0.0019	0.74	0.96	0.98
	20%	0.0025	0.0033	0.0030	0.69	0.91	0.95
	40%	0.0032	0.0044	0.0042	0.60	0.73	0.74
	80%	0.0041	0.0053	0.0052	0.53	0.52	0.53
	100%	0.0042	0.0067	0.0070	0.53	0.52	0.53

[1] PHX_0 = Unregularized PHOENIX where $\lambda_{\text{prior}} = 0$ (ignoring the network prior altogether).

[2] PHX_+ = Regularized PHOENIX where λ_{prior} was chosen based on the validation set, but both activating and repressive edges in the prior were set to +1. "No prior interaction" was kept to 0. This emulated a setting where we didn't have prior knowledge of these signs (either activating vs repressive prior edge).

[3] PHX = Regularized PHOENIX where λ_{prior} was chosen based on the validation set. Each entry in the prior was correctly set to +1 or -1, depending on whether the prior edge was activating or repressive. "No prior interaction" was set to 0.

Additional File 1: Table S4 Benchmarking PHOENIX against other methods *in silico*, in terms of trajectory-recovery performance (performance metric is MSE on test set)

		Trajectory based				Velocity based		
	Noise	PHX[1]	PHX_0[2]	OOTB[3]	PRESC[4]	Dynamo[5]	RNODE[6]	DeepVelo[7]
SIM350	0%	0.0009	0.0009	0.0027	0.0046	0.0032	0.0052	0.0062
	5%	0.0011	0.0010	0.0029	0.0043	0.0034	0.0074	0.0083
	10%	0.0014	0.0019	0.0029	0.0045	0.0040	0.0084	0.0086
	20%	0.0028	0.0025	0.0028	0.0048	0.0052	0.0104	0.0122
	40%	0.0037	0.0031	0.0042	0.0061	0.0066	0.0098	0.0093
	80%	0.0050	0.0039	0.0052	0.0066	0.0074	0.0090	0.0108
	100%	0.0059	0.0045	0.0061	0.0065	0.0080	0.0095	0.0151
SIM690	0%	0.0011	0.0009	0.0033	0.0046	0.0042	0.0076	0.0075
	5%	0.0012	0.0018	0.0029	0.0051	0.0057	0.0084	0.0074
	10%	0.0019	0.0024	0.0035	0.0057	0.0063	0.0083	0.0086
	20%	0.0030	0.0025	0.0032	0.0056	0.0061	0.0109	0.0099
	40%	0.0042	0.0032	0.0046	0.0055	0.0077	0.0100	0.0100
	80%	0.0052	0.0041	0.0058	0.0126	0.0107	0.0146	0.0103
	100%	0.0070	0.0042	0.0071	0.0133	0.0099	0.0183	0.0108

[1]PHX = Regularized PHOENIX where λ_{prior} was chosen based on the validation set.

[2]PHX_0 = Unregularized PHOENIX where $\lambda_{\text{prior}} = 0$ (ignoring the network prior).

[3]OOTB = Out-of-the-box NeuralODE, resembling how plain NeuralODEs are typically used for this problem. We note that the method RNAForecaster uses an OOTB approach with ReLU activation, but the results here are for the *tanh* activation function that had better performance than both sigmoid and ReLU on the validation set.

[4]PRESC = PRESCIENT, where we used the validation set to optimize the value of k_{dim}, which is the number of neurons use in PRESCIENT's hidden layer.

[5]For Dynamo we used the validation set to optimize the sparsity regularization penalty (λ), as well as the number of kernel basis functions use to approximate the vector field (M)

[6]RNODE = RNA-ODE, where we used the validation set to optimize the number of trees used in the random forest function

[7]In DeepVelo the encoder and the decoder consisted of four dense layers (size $4p$ for the intermediate layers and size p for the latent layer) with ReLU activation. We used the validation set to optimize p.

Additional File 1: Table S5 Benchmarking PHOENIX against other methods *in silico*, in terms of explainability. A GRN describing the inferred dynamics was extracted for each fitted model, and was compared to the ground truth GRN to calculate an AUC

		Trajectory based				Velocity based		
	Noise	PHX[1]	PHX_0[1]	OOTB[2]	PRESC[3]	Dynamo[4]	RNODE[5]	DeepVelo[6]
SIM350	0%	0.987	0.942	0.580	N/A	0.784	0.683	0.768
	5%	0.980	0.899	0.625	N/A	0.776	0.659	0.744
	10%	0.979	0.858	0.606	N/A	0.762	0.593	0.704
	20%	0.943	0.766	0.543	N/A	0.741	0.599	0.732
	40%	0.761	0.606	0.486	N/A	0.717	0.596	0.751
	80%	0.538	0.552	0.473	N/A	0.667	0.607	0.724
	100%	0.520	0.541	0.452	N/A	0.646	0.606	0.701
SIM690	0%	0.986	0.847	0.694	N/A	0.733	0.730	0.822
	5%	0.984	0.800	0.599	N/A	0.710	0.634	0.768
	10%	0.983	0.744	0.580	N/A	0.707	0.614	0.768
	20%	0.953	0.686	0.557	N/A	0.698	0.523	0.784
	40%	0.737	0.602	0.450	N/A	0.652	0.455	0.729
	80%	0.531	0.531	0.437	N/A	0.644	0.476	0.732
	100%	0.529	0.525	0.460	N/A	0.625	0.395	0.729

[1]The architecture of PHOENIX allowed GRNs to be extracted from both regularized and unregularized versions using a very simple and efficient algorithm (see Additional File 2: Section 2)

[2]GRNs were extracted from out-of-the-box NeuralODEs, using sensitivity analyses (see Additional File 2: Section 3.2)

[3]PRESCIENT does not provide a straightforward means for extracting a GRN describing the dynamics

[4]Dynamo provides a function that calculates the Jacobian matrix of the fitted model at any data point. We used this function to calculate an average Jacobian matrix across several randomly generated data points and estimate a GRN (see Additional File 2: Section 3.3)

[5]RNA-ODE provides a function for GRN inference by ranking regulatory links using estimated effect sizes (see Additional File 2: Section 3.3)

[6]DeepVelo provides a function for estimating gene correlation networks from simulated retrograde trajectories. We generated $n = 200$ retrograde trajectories using 200 randomly generated initial conditions (see Additional File 2: Section 3.3)

Additional File 1: Table S6 Benchmarking PHOENIX against other methods *in silico*, in terms of sparsity of inferred dynamics. Sparsity was calculated here as the average out-degree of the extracted GRN from each of the inferred dynamical systems

		Trajectory based				Velocity based		
	Noise	PHX	PHX_0	OOTB	PRESC[1]	Dynamo	RNA-ODE	DeepVelo
SIM350	0%	3	10	81	N/A	94	97	37
	5%	4	15	50	N/A	65	48	38
	10%	8	23	41	N/A	113	23	40
	20%	11	45	80	N/A	109	75	82
	40%	39	43	34	N/A	178	76	64
	80%	16	23	1	N/A	265	73	35
	100%	96	66	72	N/A	272	78	66
SIM690	0%	4	29	144	N/A	218	147	79
	5%	5	44	109	N/A	205	54	85
	10%	12	64	130	N/A	171	48	108
	20%	13	86	138	N/A	254	127	149
	40%	58	166	2	N/A	73	43	170
	80%	77	183	689	N/A	402	35	131
	100%	205	413	4	N/A	501	21	63

[1] PRESCIENT does not provide a straightforward means for extracting a GRN that describes the dynamics

Additional File 1: Table S7 Details about additional black-box methods for estimating gene expression dynamics that we excluded from our *in silico* benchmarking experiments. We provide details about each method, and some reasoning behind its exclusion

Method	Approach for estimating dynamics	Notes on exclusion
PROB	Uses a local Euler approximation to calculate RNA velocity $\boldsymbol{dx}/dt$. Then describes this RNA velocity as a function of gene-expression through a linear model with quadratic interaction terms between all pairs of genes. Bayesian Lasso is used to induce sparsity.	The linear model is too simplistic, and may not accurately reflect complex regulatory patterns. Also, the method seeks to directly estimate pairwise gene effects ($\mathcal{O}(n^2)$ parameters), and hence has not been shown to scale beyond 100 genes.
LatentVelo	Embeds spliced and unspliced RNA counts into a low dimensional latent space using a variational autoencoder (VAE), and then infers dynamics in this latent space with a NeuralODE, with soft constraints on the interplay between latent spliced and latent unspliced counts.	Dynamics can only be inferred in a low-dimensional latent space, making it difficult to derive interpretable insights and compare explainability. We already benchmarked against a VAE-based model (DeepVelo) that - unlike LatentVelo - provides a GRN interpreting the inferred dynamics.
scTour	Embeds spliced RNA counts into a low dimensional latent space using a variational autoencodeer, and then infers dynamics in this latent space with a NeuralODE.	Same reasoning as above.
PBA	Uses spectral-graph theory to solve multi-dimensional Fokker-Plank equations on an empirical grid formed by expression values. It assumes that velocity fields are gradients of a potential landscape in gene expression space $\boldsymbol{J} = -\nabla F$.	PBA ostensibly ignores oscillatory gene expression dynamics (the cell cycle), and hence may not be flexible enough. Also, we already benchmarked against another method (PRESCIENT), that assumes dynamics to be the gradient of a scalar potential.
PathReg	Uses an out-of-the-box NeuralODE with an Exponential Linear Unit (ELU) activation function and enforces both weight and feature sparsity at the path-level via a differentiable L_0-based regularizer in the loss function. A matrix of non-negative stochastic gates regularizes the probability of any path throughout the entire network contributing to a given output. This constrains the number of input-output paths.	PathReg was primarily developed to induce sparsity in NeuralODEs, and is similar in principle to the out-of-the-box models that we already benchmarked. Consequently, is has only been demonstrated to scale up to 529 highly variable dimensions/genes.

Additional File 1: Table S8 Qualitative comparison of black-box methods for estimating gene expression dynamics that we benchmarked against PHOENIX *in silico*. We provide details about inputs, learning algorithms, and key performance metrics

			**Scalability**	**Explainability**		**Notes**
	**Method**	**Description of approach for estimating dynamical system**	Demonstrated performance on $> 10^4$ genes without dimension reduction	Can extract GRN that describes dynamics	Flexibly incorporates prior/domain knowledge	✳ Hyperparameters we optimized using val. set<br>✂ GRN extraction details
Velocity based (two step)	Dynamo	Can use multiple modalities of seq data to estimate RNA velocity using both deterministic and stochastic approaches, then fits a sparse vector field mapping expression to velocity using Gaussian kernel regression	✘<br>(Demonstrated on $< 10^4$ genes using dimension reduction via PCA/UMAP)	✓	✘	✳ Control points (M) and sparsity parameter (λ)<br>✂ Monte Carlo approach to estimate GRN using Jacobians provided by tool
	RNA-ODE	Uses scRNA transcriptome and RNA velocity to fit random forests mapping expression to velocity	✘<br>(Demonstrated on 3001 genes)	✓	✘	✳ Number of trees (nTrees)<br>✂ Tool provides GRN
	DeepVelo	Uses mRNA counts and corresponding and RNA velocity (from scVelo) to fit variational autoencoders mapping expression to velocity	✘<br>(Demonstrated on 3000 highly variable genes)	✓	✘	✳ Number of neurons<br>✂ Tool provides gene correlation matrix by simulating retrograde trajectories
Traj. based (one step)	PRESCIENT	Uses time-series scRNA-seq and cell-growth rate data to learn a potential function Ψ with a neural network. Final drift model is obtained using automatic differentiation $\mu = -\nabla\Psi$	✘<br>(Demonstrated on 2500 highly variable genes or on top$K$ PC)	✘	✘	✳ Number of neurons inside each hidden layer (k_{dim})
	Out-of-the-box NeuralODE	Uses time-series expression and NeuralODE out-of-the-box, with traditional activation functions	✘<br>(Demo. on 2000 high expressed genes)	✓	✘	✂ Monte Carlo approach using sensitivity analysis
	PHOENIX	Our method	✓	✓	✓	✳ Prior weight (λ_{prior})<br>✂ Extraction algo.

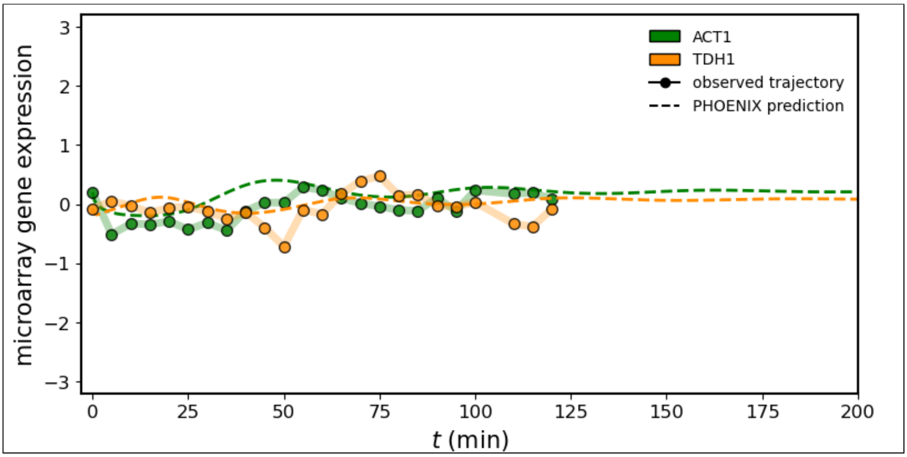

Additional File 1: Figure S6 We applied PHOENIX ($\lambda_{prior} = 0.05$) to 2 technical replicates of gene expression of 3551 genes each, collected across 24 time points in a yeast cell-cycle time course. We trained on 40 transition pairs, used 3 for validation, and tested predictive accuracy on the remaining 3. We display both observed and predicted trajectories for ACT1 and TDH1, where the predicted trajectories are extrapolations into future time points based on just initial values (gene expression at $t = 0$).

Additional File 1: Table S9 PHOENIX uses gene-specific multipliers $\boldsymbol{\upsilon} \in \mathbb{R}^n$ (see Model Formulation in main paper) to simplify the representation of steady state for genes without upstream transcription factors $\left(\frac{dg_i(t)}{dt} = 0, \forall t\right)$. Below we report the number of genes for which $\upsilon_i \leq 0$ (and hence gene i has a flat trajectory) for the PHOENIX model fitted on the **yeast cell cycle data**.

Dataset	Number of genes modelled	Number of genes i such that $\upsilon_i \leq 0$
Yeast	3551	114

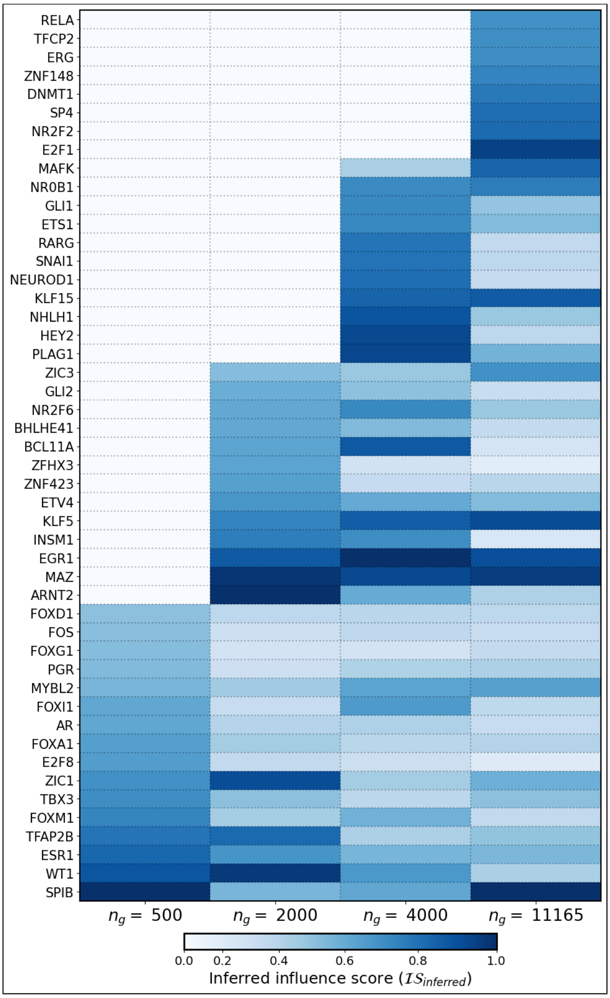

Additional File 1: Figure S7 We applied PHOENIX to a pseudotrajectory of 186 breast cancer samples (ordered along subsequent "pseudotimepoints") consisting of $n_g = 11165$ genes. We also repeated the analysis on smaller subsets of genes $n_g = 500, 2000, 4000$, where we subsetted the full trajectory to only the n_g most variable genes in the pseudotrajectory. We used the trained PHOENIX models to extract influence scores for individual genes in the estimated system (see Methods 3.3), and visualized influence scores for the most central genes across different values of n_g. For visualization purposes, the influence scores are normalized within each column (each value of n_g) to be between 0 and 1. Genes that are excluded from the subset of n_g most variable genes were assigned $\mathcal{IS}_{inferred} = 0$.

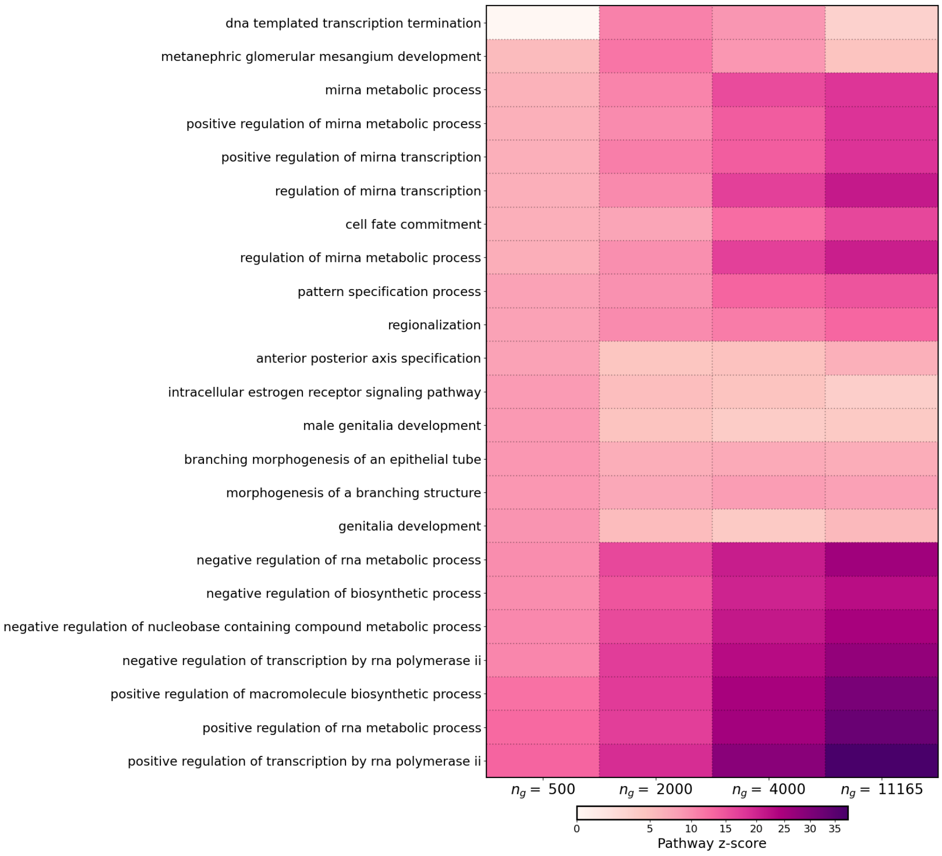

Additional File 1: Figure S8 We applied PHOENIX to a pseudotrajectory of 186 breast cancer samples (ordered along subsequent "pseudotimepoints") consisting of $n_g = 11165$ genes. We also repeated the analysis on smaller subsets of genes $n_g = 500, 2000, 4000$, where we subsetted the full trajectory to only the n_g most variable genes in the pseudotrajectory. We used the trained PHOENIX models to extract influence scores for pathways in the **Gene Ontology (biological process)** database (see Methods 3.4), and visualized influence scores for the most central pathways across different values of n_g.

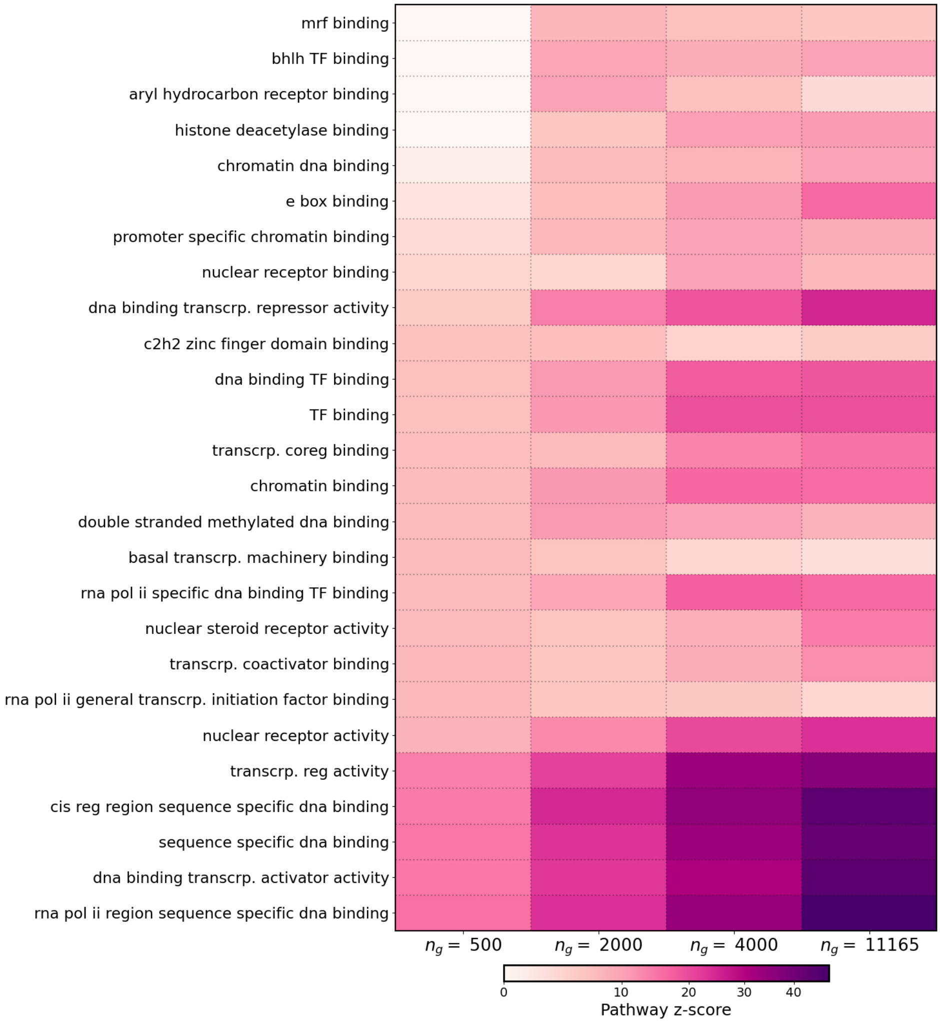

Additional File 1: Figure S9 We applied PHOENIX to a pseudotrajectory of 186 breast cancer samples (ordered along subsequent "pseudotimepoints") consisting of $n_g = 11165$ genes. We also repeated the analysis on smaller subsets of genes $n_g = 500, 2000, 4000$, where we subsetted the full trajectory to only the n_g most variable genes in the pseudotrajectory. We used the trained PHOENIX models to extract influence scores for pathways in the **Gene Ontology (molecular function)** database (see Methods 3.4), and visualized influence scores for the most central pathways across different values of n_g.

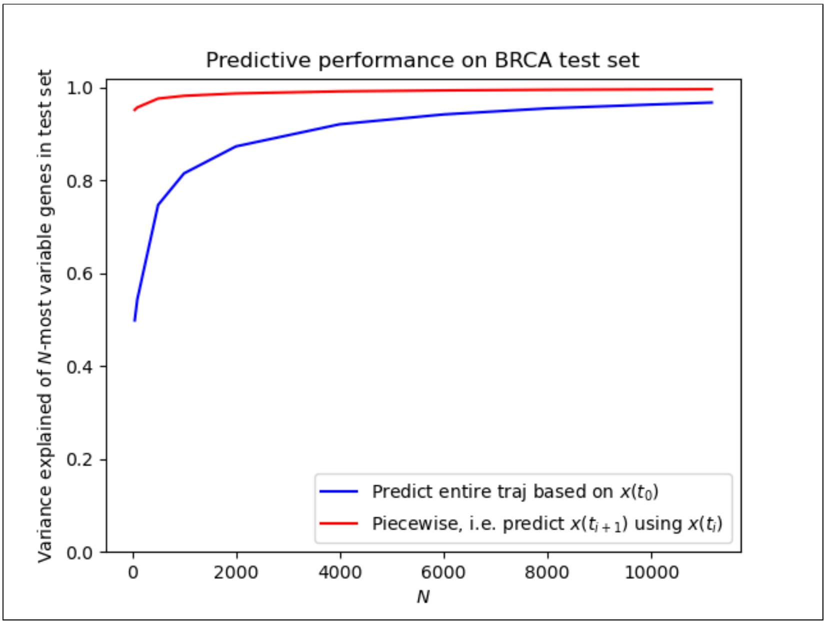

Additional File 1: Figure S10 For the breast cancer data, we depict the R^2 performance (y-axis) of a PHOENIX model trained on all genes, when considering only the N most variable genes (x-axis) for evaluation. We further evaluate the impact of predicting the entire trajectory based on the initial gene expression value (t_0, blue) versus predicting each expression value based on its immediately previous time point (red).

Additional File 1: Table S10 Detailed results of permutation tests (see Methods 3.4) used to obtain pathway influence scores from trained PHOENIX models and the **Reactome pathway database**. The mean (μ_0) and standard deviation (σ_0) of each permutation test null distribution, over $K = 1000$ permutations, are tabulated for each subset (n_g) in question, and the corresponding z-scores are visualized in **Figure 5**. Missing values indicate that the **all** genes involved in that pathway were excluded from that particular subset based on low variability in expression. Reactome accession IDs for all the pathways below have been provided in Additional File 1: Table S11 for reproducibility.

**Reactome pathway**	$n_g = 500$ genes			$n_g = 2000$ genes			$n_g = 4000$ genes			$n_g = 11165$ genes		
	z	μ_0	σ_0	z	μ_0	σ_0	z	μ_0	σ_0	z	μ_0	σ_0
sumoylation of intracellular receptors	8.001	0.008	0.007	8.055	0.003	0.002	11.678	0.009	0.003	13.834	0.003	<9e-4
nuclear receptor transcription pathway	7.81	0.013	0.009	10.956	0.007	0.002	19.355	0.017	0.004	24.588	0.005	<9e-4
estrogen dependent gene expression	6.927	0.03	0.014	8.144	0.011	0.003	15.315	0.015	0.005	15.5	0.008	0.001
transcriptional regulation by RUNX2	6.751	0.009	0.007	5.59	0.01	0.003	7.645	0.018	0.005	6	0.01	0.001
sumoylation of transcription factors	5.967	0.002	0.004	7.791	0.002	0.001	3.754	0.004	0.002	5.944	0.002	<9e-4
RUNX1 reg. wnt signaling	5.811	0.002	0.004	5.429	0.001	0.001	4.787	0.001	0.001	4.105	<9e-4	<9e-4
ESR mediated signaling	5.8	0.041	0.015	2.85	0.019	0.004	3.6	0.029	0.006	4.237	0.014	0.001
RUNX1 reg. estrogen receptor mediated transcrip.	5.692	0.003	0.004	5.518	0.001	0.001	5.649	0.001	0.001	4.138	<9e-4	<9e-4
transcriptional regulation of testis differentiation	5.618	0.004	0.004	4.317	0.003	0.001	7.503	0.006	0.003	3.375	0.001	<9e-4
regulation of RUNX2 expression and activity	5.459	0.005	0.005	5.002	0.004	0.002	5.037	0.008	0.003	3.227	0.006	0.001
nuclear signaling by ERBB4	5.371	0.008	0.007	2.436	0.004	0.002	1.46	0.01	0.004	0.731	0.003	<9e-4
RUNX2 reg. bone development	4.671	0.002	0.004	6.85	0.003	0.002	8.44	0.006	0.003	7.674	0.003	<9e-4
NGF stimulated transcription	4.238	0.007	0.006	5.484	0.007	0.002	6.593	0.01	0.004	9.107	0.003	<9e-4
transcriptional regulation of granulopoiesis	1.948	0.002	0.004	5.556	0.003	0.001	9.688	0.008	0.003	13.092	0.003	<9e-4
estrogen dep. nucl. events (downstr. ESR signal)	1.445	0.01	0.008	0.257	0.004	0.002	0.437	0.006	0.003	13.961	0.002	<9e-4
regulation of gene expression in beta cells	1.065	0.002	0.004	2.342	0.001	<9e-4	8.107	0.004	0.002	6.665	0.001	<9e-4
regulation of pten gene transcription	-0.307	0.003	0.004	5.367	0.001	0.001	7.716	0.009	0.004	4.028	0.005	0.001
transcriptional regulation of pluripotent stem cells	-0.474	0.003	0.004	3.309	0.003	0.001	6.545	0.007	0.003	10.134	0.002	<9e-4
aryl hydrocarbon receptor signalling				9.454	0.001	0.001	6.065	0.001	0.001	2.482	0.001	<9e-4
RUNX2 reg. chondrocyte maturation				7.833	0.001	0.001	6.172	0.001	0.001	4.482	<9e-4	<9e-4
Xenobiotics				6.516	0.001	0.001	2.456	0.005	0.003	2.145	0.001	<9e-4
GLI proteins bind promoters of Hedgehog				6.378	0.001	0.001	8.154	0.003	0.002	6.22	<9e-4	<9e-4
RUNX3 reg. CDKN1A transcription				5.776	0.001	0.001	2.117	0.001	0.001	5.43	0.001	<9e-4
suppression of apoptosis				-0.04	0.001	0.001	0.094	0.001	0.001	10.147	0.001	<9e-4
myogenesis				-0.223	0.001	0.001	1.283	0.004	0.002	7.772	0.002	<9e-4
TP53 reg. apoptosis				-0.295	0.002	0.001	4.796	0.004	0.002	13.769	0.001	<9e-4
TP53 reg. transcrip. of caspase activators							8.13	0.001	0.001	3.15	0.001	<9e-4
MECP2 reg. transcription factors							4.4	0.001	0.001	9.114	<9e-4	<9e-4

Additional File 1: Table S11 Accession IDs for Reactom pathways described in **Figure 5** (main paper) and Additional File 1: Table S10. We shortened the names of pathways to fit them into tables and figures throughout the paper and supplement. Hence we're providing the accession IDs here.

**Pathway**	**Reactome Stable Identifier**
Sumoylation of intracellular receptors	R-HSA-4090294
Nuclear receptor transcription pathway	R-HSA-383280
Estrogen dependent gene expression	R-HSA-9018519
Transcriptional regulation by RUNX2	R-HSA-8878166
Sumoylation of transcription factors	R-HSA-3232118
RUNX1 reg. wnt signaling	R-HSA-195721
ESR mediated signaling	R-HSA-8939211
RUNX1 reg. estrogen receptor mediated transcrip.	R-HSA-8931987
Transcriptional regulation of testis differentiation	R-HSA-9690406
Regulation of RUNX2 expression and activity	R-HSA-8939902
Nuclear signaling by ERBB4	R-HSA-1251985
RUNX2 regulates bone development (corrected)	R-HSA-8941326
NGF stimulated transcription	R-HSA-9031628
Transcriptional regulation of granulopoiesis	R-HSA-9616222
Estrogen dep. nucl. events (downstr. ESR signal)	R-HSA-9634638
Regulation of gene expression in beta cells	R-HSA-210745
Regulation of pten gene transcription	R-HSA-8943724
Transcriptional regulation of pluripotent stem cells	R-HSA-452723
Aryl hydrocarbon receptor signaling	R-HSA-8937144
RUNX2 reg. chondrocyte maturation	R-HSA-8941284
Xenobiotics	R-HSA-211981
GLI proteins bind promoters of Hedgehog	R-HSA-5635851
RUNX3 reg. CDKN1A transcription	R-HSA-8941855
Suppression of apoptosis	R-HSA-9635465
Myogenesis	R-HSA-525793
TP53 reg. apoptosis	R-HSA-5633008
TP53 reg. transcrip. of caspase activators	R-HSA-6803207
MECP2 reg. transcription factors	R-HSA-9022707

Additional File 1: Table S12 PHOENIX uses gene-specific multipliers $\boldsymbol{\upsilon} \in \mathbb{R}^n$ (see Model Formulation in main paper) to simplify the representation of steady state for genes without upstream transcription factors $\left(\frac{dg_i(t)}{dt} = 0, \forall t\right)$. Below we report the number of genes for which $\upsilon_i \leq 0$ (and hence gene i has a flat trajectory) for each of the PHOENIX models fitted on the **breast cancer data**.

Dataset	Number of genes modelled (n_g)	Number of genes i such that $\upsilon_i \leq 0$
BRCA	500	14
BRCA	2000	161
BRCA	4000	138
BRCA	11165	289

Additional File 1: Table S13 Additional performance metrics for PHOENIX on breast cancer data

Number of genes (n_g)[1]	Runtime (hrs)[2]	Concordance with influential ChIP genes[3]
500	0.10	11.48%
2000	0.14	18.03%
4000	0.50	31.15%
11165	2.52	80.33%

[1]The full data set consisted of $n_g = 11165$ genes. We also fit PHOENIX to smaller subsets of genes $n_g = 500, 2000, 4000$, where we subsetted the full data set to only the n_g most variable genes in the pseudotrajectory.

[2]Runtime for a single run when using an AWS c5.4xlarge instance ($0.68/hour).

[3]For each n_g, we computed inferred influence scores for individual genes based on perturbation analyses on the fitted PHOENIX model (see Methods 3.3). We also computed harmonic centralities of the genes based on a validation network describing ChIP-binding data. We then did a binary assignment, where the top 10% ($10\% \times n_g$) genes with highest inferred influence were labelled "predicted influential" and the remaining ($90\% \times n_g$) were "predicted non-influential." We measured concordance as the **sensitivity** $\frac{TP}{TP+FN}$ with which these predicted labels recovered the "truly influential" genes (those genes with non-zero harmonic centrality in the ChIP validation network).

Additional File 1: Table S14 We compared the performance of PHOENIX to other methods of regulatory dynamics estimation on a pseudotrajectory of 186 breast cancer samples (ordered along subsequent "pseudotimepoints") consisting of $n_g = 11165$ genes. The data was processed for model fitting via the steps described in Methods 3.2. Snapshot-based methods (Dynamo, RNA-ODE, Deepvelo) require RNA velocity at every time point as an additional input. Given that this information was not available in the data set, we estimated RNA velocity using a method of finite differences applied to smooth splines through the expression trajectories (see Additional File 2: Section 3.1). Once each model was trained, we measured predictive accuracy in a step-wise fashion; for N ranging from 50 to 11165, we calculated the **test set MSEs** (as described in Methods 3.2) of only the top-N most varying genes in the dataset.

N[1]	Dynamo	RNA-ODE	PHOENIX	DeepVelo	OOTB[2]
50	1.61E-02	1.60E-02	**1.57E-02**	1.68E-02	1.59E-02
100	1.22E-02	1.21E-02	**1.18E-02**	1.20E-02	1.20E-02
500	5.76E-03	5.73E-03	5.62E-03	5.81E-03	**5.54E-03**
1000	4.04E-03	4.02E-03	3.95E-03	4.95E-03	**3.90E-03**
2000	2.78E-03	2.77E-03	**2.72E-03**	2.83E-03	2.79E-03
4000	1.79E-03	1.78E-03	**1.75E-03**	**1.75E-03**	1.77E-03
6000	1.32E-03	1.32E-03	**1.29E-03**	1.30E-03	1.30E-03
8000	1.05E-03	1.04E-03	**1.02E-03**	1.07E-03	1.03E-03
10000	8.58E-04	8.56E-04	**8.39E-04**	8.44E-04	8.43E-04
11165	7.73E-04	7.70E-04	**7.55E-04**	7.76E-04	7.59E-04

[1]N = Number of most highly variable genes being considered in the MSE calculation.

[2]OOTB = Out-of-the-box NeuralODE, resembling how plain NeuralODEs are typically used for this problem.

Additional File 1: Table S15 Top 50 most changing regulators discovered by PHOENIX in B cells after Rituximab treatment as measured by log-fold change.

Gene Symbol	log-fold change	Gene Symbol	log-fold change
PNRC2	2.56873290336475	PPP2R3C	2.47535285573376
PCDHAC2	2.4183531041133	PSD	-2.33736834391816
SDF2L1	-2.32960158182622	PHLPP2	-2.31911018375466
TTC27	2.31393595893796	SLFN12	2.31373381881861
HMOX2	2.29844754939876	ARPC5L	2.28379510626964
EXOSC9	2.27076773960713	CCT6A	2.26711838782967
CLUH	-2.26276854159785	NACC2	-2.25002672061429
CCDC51	2.23323817802628	ACAA2	2.23302148320103
RNF10	2.2271584454618	CCNT1	2.22571736590982
GRAP	-2.2202087315781	TIMM8B	-2.20914724136027
GOLGA4	2.20154634635607	DTNBP1	2.16880863093615
SETBP1	-2.16869943368544	GNG10	-2.16748492186343
ASL	2.16732773542853	HNRNPA1L2	-2.16278244412603
MRPS5	-2.15142782602203	COA6	2.15119270022043
NOL8	-2.14932979658522	NKAP	2.14646756016082
SMYD4	2.14241657408929	TMEM181	2.14176930774647
PPM1K	2.14122503727424	TMEM43	-2.13977794610878
ZC3H4	2.13435569243602	UPRT	-2.12251533684904
TMEM9B	-2.12219325230208	WIPI1	-2.11544001910886
ATP1A1	-2.10439941185222	ISCU	-2.10262448389575
TOX4	2.10142807192934	ABI2	-2.09719154828222
AGGF1	2.09364544925298	KTI12	-2.09231153185146
CHRDL2	-2.08727487121291	HACL1	-2.0871444586354
CNTN4	-2.08664483437801	MAML1	-2.08413955022521
RAB1A	-2.08379529871721	MFNG	2.07967407563063

Additional File 1: Table S16 Training results from PHOENIX models applied to the B-cell RNASeq datasets. We fitted two seperate PHOENIX models, one for each condition of untreated and treated with Rituximab. Furthermore, PHOENIX uses gene-specific multipliers $\boldsymbol{\upsilon} \in \mathbb{R}^n$ (see Model Formulation in main paper) to simplify the representation of steady state for genes without upstream transcription factors $\left(\frac{dg_i(t)}{dt} = 0, \forall t\right)$; so we report the number of genes for which $\upsilon_i \leq 0$ (and hence gene i has a flat trajectory) for each of two models.

Condition	#Genes modelled	val. set MSE	train set R^2	#Genes with $\upsilon_i \leq 0$
Untreated	14691	0.0015	91.77%	186
Treated	14691	0.0021	89.58%	167

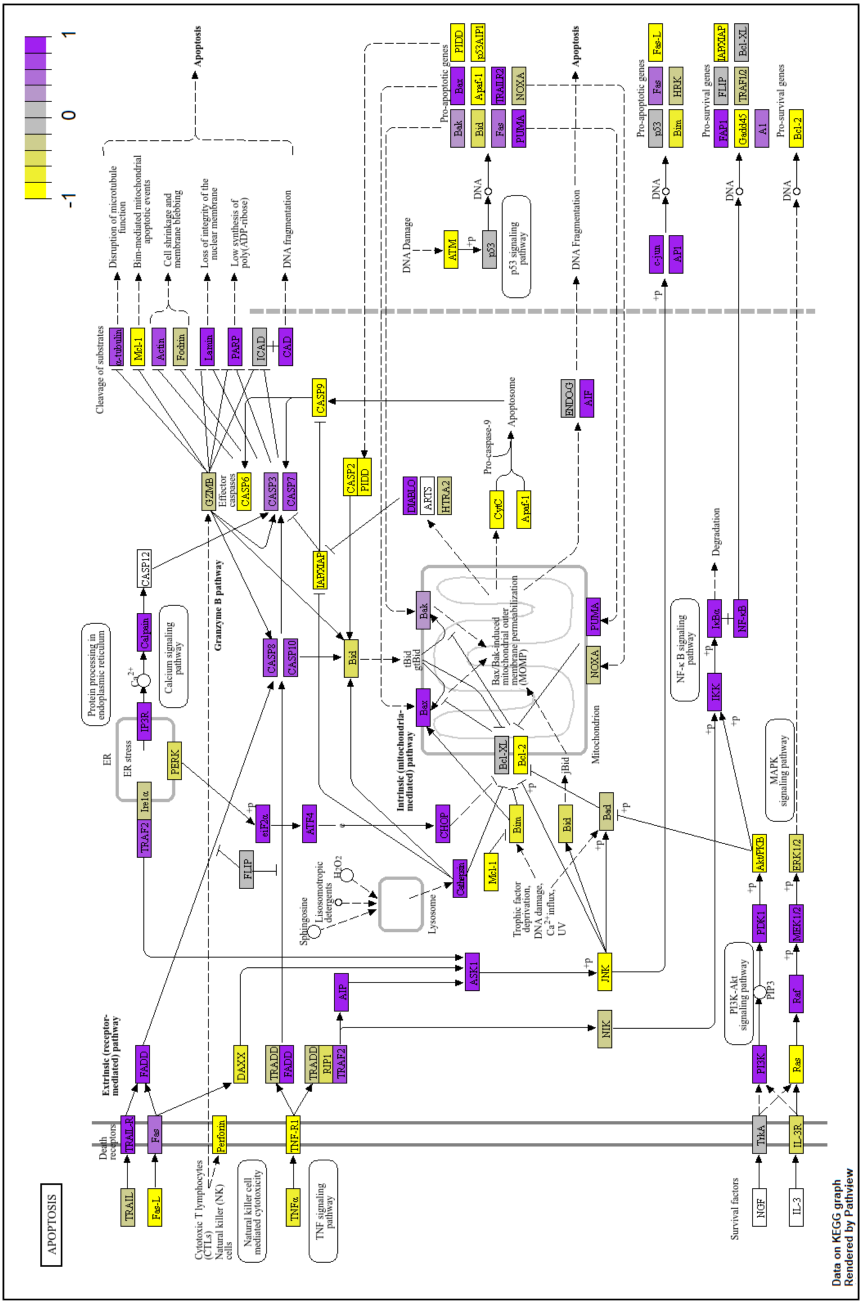

Additional File 1: Figure S11 Regulatory changes discovered by PHOENIX in B cells after Rituximab treatment. All genes annotated in the KEGG **Apoptosis pathway** are colored by log-fold change between treatment and control, the range of values has been normalized to $[-1, 1]$. Positive values/purple color shades mean that these genes show higher regulatory influence in Rituximab-treated B cells compared to control.

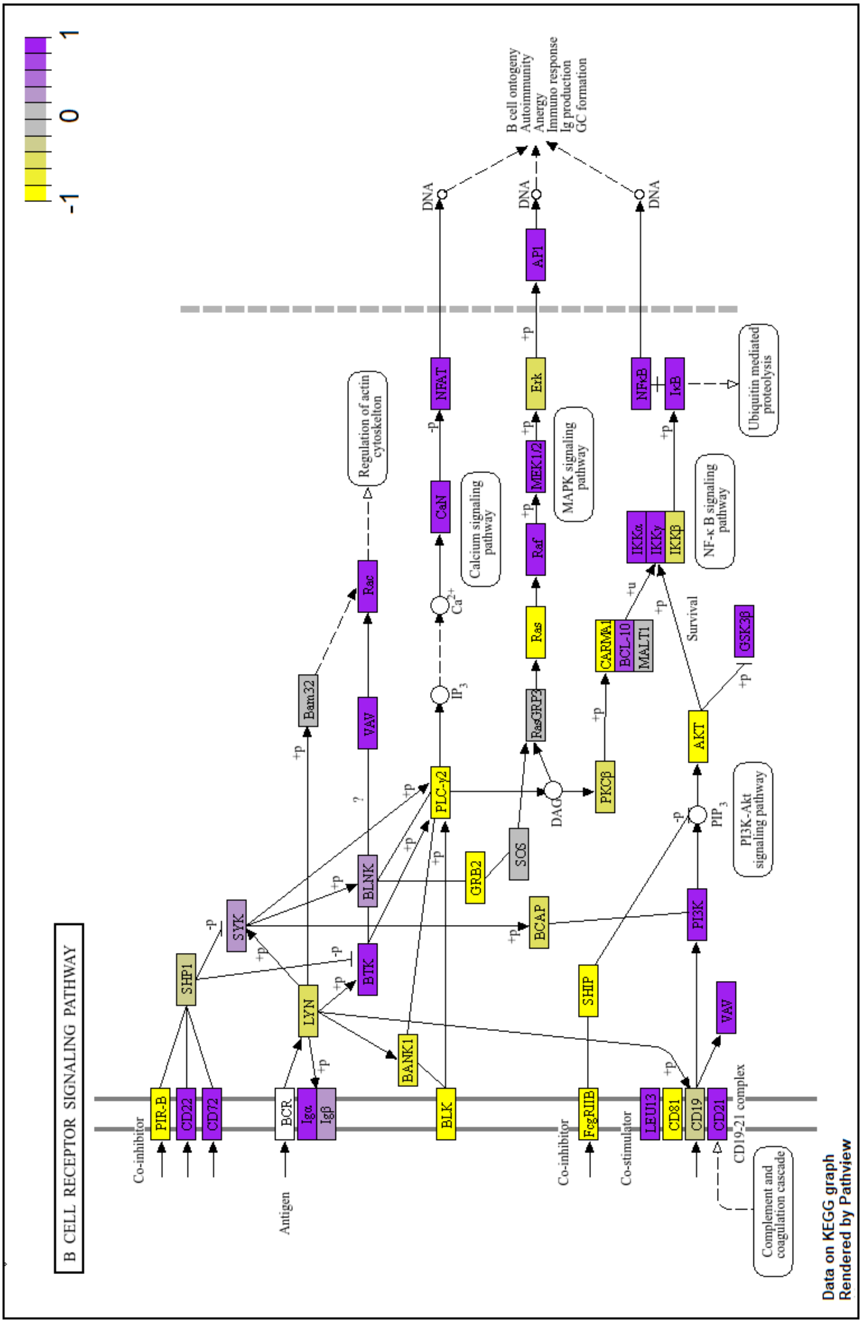

Additional File 1: Figure S12 Regulatory changes discovered by PHOENIX in B cells after Rituximab treatment. All genes annotated in the KEGG **B cell receptor signaling pathway** are colored by log-fold change between treatment and control, the range of values has been normalized to $[-1, 1]$. Positive values/purple color shades mean that these genes show higher regulatory influence in Rituximab-treated B cells compared to control.
