# Supplementary material for: Biologically informed NeuralODEs for genome-wide regulatory dynamics
Source: Genome Biol. 2024 May 21;25:127. doi: 10.1186/s13059-024-03264-0 (PMC11106922; doi:10.1186/s13059-024-03264-0)
Supplement: Supplementary file 2 — Additional file 2: Supplemental methods. This file contains additional implementation and methodological details of PHOENIX, as well as implementation details of competing methods for the benchmarking experiments. [file 13059_2024_3264_MOESM2_ESM.pdf]

Supplemental methods accompanying: "Biologically informed neural ordinary differential equations for genome-wide regulatory dynamics"

Intekhab Hossain[1*], Viola Fanfani[1], Jonas Fischer[1], John Quackenbush[1] and Rebekka Burkholz[2]

[1*]Department of Biostatistics, Harvard T.H. Chan School of Public Health, Boston, MA, USA.
[2]Helmholtz Center for Information Security (CISPA), Saarbrücken, Germany.

*Corresponding author(s). E-mail(s): ihossain@g.harvard.edu;
Contributing authors: vfanfani@hsph.harvard.edu; jfischer@hsph.harvard.edu; johnq@hsph.harvard.edu; burkholz@cispa.de;

This is the supplementary methods file accompanying the paper. For further queries, please feel free to reach out to `ihossain@g.harvard.edu`

Additional File 2: Section 1 PyTorch: details

Additional File 2: Section 1.1 NN architecture and forward()

Both NN_{sums} and NN_{prods} are fully connected single layer NNs with Hill-like activations that take n inputs (for n genes) and produce m outputs each.

```
(NN_sums): Sequential(
    (activation_0): phi_Sigma()
    (sum_combos): Linear(in_features=n,out_features=m, bias = True))

(NN_prods): Sequential(
    (activation_0): phi_Pi()
    (prod_combos): Linear(in_features=n,out_features=m, bias = True))
```

We use $m << n$ allowing us to approximate lower dimensional sets of additive and multiplicative gene combinations. The choice of m is determined by:

1. We surmised that m should roughly scale with n
2. Given a value of n, we selected a plausible range of values for m, and picked m based on the predictive performance on the validation set. This allows for a means to balance between fitting accuracy and risk of overfitting.

This lead to the following size m in each experiment, where m roughly scaled with the dimensionality n of the problem,

- Simulations: For ($n = 350, 690$ genes) we use $m = 40, 50$, respectively
- Yeast cell cycle: There were $n = 3551$ genes, and we used $m = 120$
- Breast cancer: For subset sizes $n_g = 500, 2000, 4000, 11165$ genes we used $m = 40, 100, 120, 300$ respectively
- B-cell RNASeq: There were $n = 14691$ genes, and we found used $m = 200$ to give the best validation performance.

These $2m$ outputs are then fed into a final fully connected single layer $\text{NN}_{combine}$, and gene-specific parameters ($\boldsymbol{v}$/ `u_vector` $\in \mathbb{R}^n$) are applied to create the `final` estimations for the n local derivatives.

```
(NN_combine): Sequential(
    (final_outputs): Linear(in_features=2*m,out_features=n))

def forward(self, t, input):
    c_Sigma = self.NN_sums(input)
    c_Pi = torch.exp(self.NN_prods(input))
    joint = self.NN_combine(torch.cat((c_Sigma, c_Pi),dim=-1)
    final = torch.relu(u_vector)*(joint - y)
    return(final)
```

Additional File 2: Section 1.2 Predictive performance: training, testing, and validation (choosing $\boldsymbol{\lambda}$)

We re-shaped the simulated data sets to be amenable with the `torchdiffeq` package in `PyTorch` [28], which contains the base library used for NeuralODEs. Regardless of whether the data was split based on trajectories (*in silico* experiments) or transition pairs (real data applications), *at any given training step* the data was fed into the PHOENIX model in pairwise-vector form. For instance, in the *in silico* experiments, each training trajectory consisted of 5 time-points $\in \{0, 2, 3, 7, 9\}$, and was subsequently fed to the model in the form of its 4 constituent transition pairs $(t_i, t_{i+1}) \in \{(0, 2), (2, 3), (3, 7), (7, 9)\}$, where a transition pair consists of two consecutive expression vectors in the trajectory $(\boldsymbol{g}(t_i),\ \boldsymbol{g}(t_{i+1}))$. With each transition pair fed to the model, it learned an approximation of the derivative that described the transition of $\boldsymbol{g}(t_i)$ to $\boldsymbol{g}(t_{i+1})$. We initialized each of NN_{sums}, NN_{prods}, and $\text{NN}_{combine}$ with a sparse initialization scheme that set 95% of the weight parameters to be 0. We initialized the $\boldsymbol{v}_i$s with i.i.d standard uniform values.

We used `dopri5` (included with `torchdiffeq`) as the ODESolver within PHOENIX's NeuralODE engine, and the Adam optimizer (also included with `torchdiffeq`) to optimize NN parameters. Broadly speaking, `torchdiffeq` learns by performing back propagation through the ODESolver via adjoint sensitivity analysis [27]. Next, we use a `simulated_batch` of expression values $\{\boldsymbol{\gamma_k}\}_{k=1}^{K}$ to incorporate a structural domain knowledge inspired prior model. Since the $\boldsymbol{\gamma_k}$s were simulated expression values, we also refer to them as "ghost" expression values.

$$\mathcal{P}^*\left(\boldsymbol{\gamma_k}\right) + \boldsymbol{\gamma_k} = \boldsymbol{A} \cdot \boldsymbol{\gamma_k}$$

So, given the user-supplied `prior_matrix` $\boldsymbol{A}$, we pre-calculated `prior_output`, $\mathcal{P}^*\left(\boldsymbol{\gamma_k}\right) + \boldsymbol{\gamma_k}$, using `torch.matmul()`.

```
simulated_batch = torch.rand(10000,1,prior_mat.shape[0])
prior_output = torch.matmul(batch_for_prior,prior_mat)
```

We tied this into model training using a modified loss function `composed_loss` with weight (λ). We obtained model `predictions` using the `odeint()` function in `torchdiffeq`, as shown in the pseudocode below:

```
def training_step(...., training_batch, target, simulated_batch,
                    prior_output, lambda, time_pts):
    predictions =  odeint(....,training_batch, time_pts)
    loss_data = torch.mean((predictions - target)**2)
    model_sim_output = forward(time_pts,simulated_batch) +
                                                simulated_batch
```

```
    loss_prior = torch.mean((model_sim_output - prior_output)**2)
    composed_loss = lambda * loss_data +
                    (1- lambda) * loss_prior
    composed_loss.backward()
    opt.step()
    return [loss_data, loss_prior]
```

We trained for up to 200 epochs on an AWS c5.9xlarge instance, where each epoch consisted of the entire training set being fed to the model in the form of constituent transition pairs. We used `torch.optim` function `ReduceLROnPlateau()` to reduce the learning rate by 10% every 3 epochs, unless the validation set performance showed reasonable improvement.

```
scheduler = optim.lr_scheduler.ReduceLROnPlateau(opt, mode='min',
            factor=0.9, patience=3, threshold=1e-05,
            threshold_mode='abs', eps=1e-09)
```

Training terminated if validation set performance failed to improve in 10 consecutive epochs. We repeated this entire pipeline for a grid of $\lambda \in \{0.1, 0.2, 0.5, 0.8, 0.9, 0.99, 0.999, 1\}$, and used the validation mean squared error (MSE) at model termination to decide on an optimal λ. For this final model, we evaluated final predictive performance using test set MSE.

For even further details (exact learning rates, `torchdiffeq` details, ODE-Solver details, optimizer details, etc.) we refer the reader to our GitHub repository [37], where the entire code base is made available.

Additional File 2: Section 2 Explainability: GRN inference

Additional File 2: Section 2.1 Algorithm for efficiently retrieving encoded GRN from trained PHOENIX model

We start with PHOENIX's prediction for the local derivative given a gene expression vector $\boldsymbol{g}(t) \in \mathbb{R}^n$ in an n-gene system:

$$\widehat{\frac{d\boldsymbol{g}(t)}{dt}} = \text{ReLU}(\boldsymbol{v}) \odot \left[\boldsymbol{W}_{\cup}\{\boldsymbol{c}_{\boldsymbol{\Sigma}}(\boldsymbol{g}(t)) \oplus \boldsymbol{c}_{\boldsymbol{\Pi}}(\boldsymbol{g}(t))\} - \boldsymbol{g}(t)\right], \quad \text{where}$$

$$\boldsymbol{c}_{\boldsymbol{\Sigma}}(\boldsymbol{g}(t)) = \boldsymbol{W}_{\boldsymbol{\Sigma}}\phi_{\Sigma}(\boldsymbol{g}(t)) + \boldsymbol{b}_{\boldsymbol{\Sigma}} \quad \text{and} \quad \boldsymbol{c}_{\boldsymbol{\Pi}}(\boldsymbol{g}(t)) = \exp\circ(\boldsymbol{W}_{\boldsymbol{\Pi}}\phi_{\Pi}(\boldsymbol{g}(t)) + \boldsymbol{b}_{\boldsymbol{\Pi}})$$

We observed that a trained PHOENIX model encodes interactions *between* genes primarily within the gene-specific multipliers $\boldsymbol{v} \in \mathbb{R}^n$, and the weight parameters from its neural network blocks $\boldsymbol{W}_{\boldsymbol{\Pi}}, \boldsymbol{W}_{\boldsymbol{\Sigma}} \in \mathbb{R}^{m\times n}$ and $\boldsymbol{W}_{\cup} \in \mathbb{R}^{n\times 2m}$. This inspired an efficient means of projecting the estimated dynamical system down to a gene regulatory network (GRN) $\widehat{G_n}$.

We first calculated a matrix $\boldsymbol{D} \in \mathbb{R}^{n\times n}$, where $\boldsymbol{D}_{ij}$ approximated the *absolute contribution* of gene j to the derivative of gene i's expression:

$$\boldsymbol{D} = \boldsymbol{W}_{\cup}\begin{bmatrix}\boldsymbol{W}_{\boldsymbol{\Sigma}}\\ \boldsymbol{W}_{\boldsymbol{\Pi}}\end{bmatrix}$$

We applied the gene-specific multipliers $\boldsymbol{v}$, before adapting the marginal attribution approach described by Hackett *et al.* [2]. This resulted in the **dynamics matrix** $\widetilde{\boldsymbol{D}}$ where $\widetilde{\boldsymbol{D}_{ij}}$ was scaled according to the *relative contribution* of gene j to the rate of change in gene i's expression:

$$\widetilde{\boldsymbol{D}_{ij}} = \frac{\boldsymbol{v}_i\boldsymbol{D}_{ij}}{\sum_{j'=1}^{n}|\boldsymbol{v}_i\boldsymbol{D}_{ij'}|}$$

Finally, we subjected $\widetilde{\boldsymbol{D}}$ to a cut-off value $v_{\mathcal{C}}$ based on an appropriate percentile $\mathcal{C}$; we used $\mathcal{C} = 0.995$, meaning $v_{\mathcal{C}} = 99.5^{th}$ percentile of $\{|\ \widetilde{\boldsymbol{D}_{ij}}\ |\}_{\forall i,j}$. Specifically, we decided edge **existence** and **strength** in $\widehat{G_n}$ as follows:

$$\textbf{No edge}: \text{gene } j \overset{0}{\rightarrow} \text{gene } i \iff |\ \widetilde{\boldsymbol{D}_{ij}}\ | \leq v_{\mathcal{C}}$$

$$\textbf{Activating edge}: \text{gene } j \overset{\widetilde{\boldsymbol{D}_{ij}}}{\rightarrow} \text{gene } i \iff \widetilde{\boldsymbol{D}_{ij}} > v_{\mathcal{C}}$$

$$\textbf{Repressive edge}: \text{gene } j \overset{\widetilde{\boldsymbol{D}_{ij}}}{\rightarrow} \text{gene } i \iff \widetilde{\boldsymbol{D}_{ij}} < -v_{\mathcal{C}}$$

Additional File 2: Section 2.2 Evaluation of explainability

We compared the estimated $\widehat{G_n}$ to the corresponding validation network in terms of out-degree correlation and edge-existence, calculating recovery AUC, true positive rate (classification TPR), and true negative rate (classification TNR). For our *in silico* experiments, we reverse-engineered a method to inform how sparsely PHOENIX had inferred the dynamics.

Since the ground truth graphs G_{350} and G_{690} were known, we found the value of $\mathcal{C} \in (0, 1)$, the aforementioned percentile cutoff of $\mid \widetilde{\boldsymbol{D}_{ij}} \mid$ values, that maximized the balanced classification accuracy $(\frac{\text{TPR}+\text{TNR}}{2})$, and used this $\mathcal{C}_{\text{max}}$ as a measure of how sparsely the dynamics were inferred. Since $\mathcal{C}_{\text{max}}$ reflects how well the trained PHOENIX model discriminates between true and non interactions, a low value of $\mathcal{C}_{\text{max}}$ corresponded to dense dynamics, while a high value corresponded to sparser dynamics. When comparing explainability across different PHOENIX fits, we used $\mathcal{C}_{\text{max}}$ to obtain corresponding values TPR_{max} and TNR_{max}. This eliminated the dependence on any arbitrary cutoff and allowed us to compare networks in terms of best possible TPR and TNR.

Additional File 2: Section 3 Benchmark experiments against existing methods

Additional File 2: Section 3.1 Data sets used for benchmarking

For comparison to OOTB models, we used the same simulated data sets from SIM350 and SIM690 that were used for the PHOENIX experiments, with the same train-val-test split. For PRESCIENT [4], we found it to work only with consecutive integer time points, and so we generated 160 noisy trajectories from the ground-truth SIM350 and SIM690 at $t \in T = \{0, 1, 2, 3, 4, 5, 6, 7, 8, 9\}$. Each of the 1600 simulated vectors in $\{\{\boldsymbol{g}(t)_k \in \mathbb{R}^n\}_{t \in T}\}_{k=1}^{160}$ could be conceptualized as a **single cell**, with `cell_id` $= (t, k)$, at time t with expression values for n genes. We partitioned the cells via the same train-val-test split as the PHOENIX runs, and used this time-labelled "single cell training data" as PRESCIENT's input. We set all `cell_type` to `regular` in the meta data.

For "two-step" methods such as Dynamo [3], RNA-ODE [5], and DeepVelo [12], we know that they estimate dynamics by first reconstructing RNA velocity using inputs such as spliced and unspliced mRNA counts (step 1) and then estimating a vector field mapping expression to velocity (step 2). To avoid the need for uncommon input data types and to also emulate the **theoretically optimal performance** in step 1, we used the noiseless ground truth velocities $\frac{d\boldsymbol{g}(t)}{dt}$ as input into step 2 directly, since these true velocities could be retrieved from our simulator based on the random seeds used. Subsequently, we only tested step 2 (with the same train, validation, and test data sets used in PHOENIX and the corresponding ground truth velocities) and obtained an optimistic estimate of each method's performance.

As just discussed above, Dynamo, RNA-ODE, and DeepVelo are "two-step" snapshot based methods that require RNA velocity at every time point as an additional input [3, 5, 12]. Given that this information was not available in either the yeast or the breast cancer data set, we estimated RNA velocity using a method of finite differences applied to smooth splines through the expression trajectories [43], in order to apply these methods to those datasets. Specifically, for each gene in the dataset, we used the local estimator of scatterplot smoothing function `loess()` in `R` to fit a smooth spline through its expression trajectory (degree = 2, smoothing parameter $\alpha = 2$). Then we simply calculated the local derivative w.r.t. time on this smooth spline using `diff(smoooth_y)/diff(t)` in `R`. Since this approach calculates a backward finite difference, we manually copied the derivative at t_2 on to t_1.

Additional File 2: Section 3.2 Out-of-the-box NeuralODE models

We tried to emulate how one might typically use OOTB NeuralODE models for the purpose of predicting gene expression dynamics [6]. Given a gene regulatory network of n genes, we assume that the gene expression of all genes $g_j(t)$ can have an effect on a specific $g_i(t)$: $\frac{d\boldsymbol{g}(t)}{dt} = f_{reg}\Big(\boldsymbol{g}(t)\Big) - \boldsymbol{g}(t)$, where $\boldsymbol{g}(t) = \{g_i(t)\}_{i=1}^{n}$ and $f_{reg} : \mathbb{R}^n \rightarrow \mathbb{R}^n$. We approximated f_{reg} with just an out-of-the-box neural network (NN_{OOTB}) with parameters $\boldsymbol{\theta}_{OOTB}$, and ReLU, tanh, or sigmoid activation functions:

$$\frac{d\boldsymbol{g}(t)}{dt} \approx \text{NN}_{OOTB}\Big(\boldsymbol{g}(t), \boldsymbol{\theta}_{OOTB}\Big) - \boldsymbol{g}(t)$$

For fair comparison, we created NN_{OOTB} such that it contained a similar number of hidden layers and trainable parameters as PHOENIX. Specifically, we designed (`basic_act_funcs` used were Sigmoid, tanh, and ReLU) as follows:

```
(NN_OOTB): Sequential(
    (activation_0): basic_act_func()
    (layer_1): Linear(in_features=n, out_features=2*m, bias=True)
    (activation_1): basic_act_func()
    (layer_out): Linear(in_features=2*m, out_features=n), bias=True)

def forward(self, t, input):
    res = self.NN_OOTB(input)
    return(res - y)
```

We chose $m = 40$ for $n = 350$ (SIM350), and $m = 50$ for $n = 690$ (SIM690). We used the same basic learning strategy as the experiments with PHOENIX. These details as well as other technicalities are on our GitHub repository [37].

To evaluate explainability, we bootstrapped the trained NN_{OOTB} to estimate the encoded GRN. We randomly generated 100 input expression vectors via i.i.d standard uniform sampling $\{\boldsymbol{b_k} \in \mathbb{R}^n\}_{k=1}^{100}$. Next, for each gene j, we created a perturbed version of these input vectors $\{\boldsymbol{b_k^j}\}_{k=1}^{100}$, where only gene j was perturbed in each vector. We then fed both sets of input vectors into the trained NN_{OOTB} to obtain corresponding perturbed output $\{\widehat{\boldsymbol{o}}_{\boldsymbol{k}}^{\boldsymbol{j}} \in \mathbb{R}^{n_g}\}_{k=1}^{200}$ and unperturbed output $\{\widehat{\boldsymbol{o}}_{\boldsymbol{k}} \in \mathbb{R}^{n_g}\}_{k=1}^{200}$. Next, for each perturbed gene j in the input, we measured how much the perturbed output of every other gene i changed, *as a proportion* of its unperturbed output. This generally yielded better results in favor of the OOTB models than if we proceeded without

taking proportions, or normalized based on input perturbation.

$$\boldsymbol{\Delta}_{ij} = \frac{1}{200} \sum_{k=1}^{100} \left| \frac{\widehat{\boldsymbol{o}}^{\boldsymbol{j}}_{\boldsymbol{k}i} - \widehat{\boldsymbol{o}}_i}{\widehat{\boldsymbol{o}}_i} \right|$$

We used $\boldsymbol{\Delta}$ to create the normalized effects matrix $\widetilde{\boldsymbol{E}}$ such that $\widetilde{\boldsymbol{E}}_{ij}$ reflected the *relative contribution* of gene j's input to the proportion change in gene i's output: $\widetilde{\boldsymbol{E}}_{ij} = \frac{\boldsymbol{\Delta}_{ij}}{\sum_{j=1}^{n} \boldsymbol{\Delta}_{ij}}$. Finally, by thresholding the values of $\widetilde{\boldsymbol{E}}$ using an optimal cut off $\mathcal{C}_{\max}$ (see Additional File 2: Section 2.2; this is the the percentile cut off of $\widetilde{\boldsymbol{E}}_{ij}$ values that maximizes $\frac{TPR+TNR}{2}$), we obtained adjacency matrices describing $\widehat{G_{350}}$ and $\widehat{G_{690}}$ that approximated the ground truth G_{350} and G_{690}, respectively.

Additional File 2: Section 3.3 Other benchmarked methods

We provide some detail here, but source code (and even more technicalities) can be found on our GitHub repository [37], where this entire benchmarking pipeline is made available. For assessing explainability in each case, we only explain up to how a matrix $\widetilde{\boldsymbol{E}}$ was obtained. We then processed $\widetilde{\boldsymbol{E}}$ in the same way as described at the end of Additional File 2: Section 3.2 to obtain $\widehat{G_n}$. We calculated $\widehat{G_n}$'s average out-degree and obtained AUC by comparing to ground truth G_n.

Additional File 2: Section 3.3.1 PRESCIENT

PRESCIENT [4] uses time-series scRNA-seq and cell-growth rate data to learn a scalar-valued potential function $\Psi(\boldsymbol{g}(t))$ with a neural network. A final drift model is obtained using automatic differentiation $\frac{\boldsymbol{g}(t)}{dt} = -\nabla\Psi(\boldsymbol{g}(t))$. We found that PRESCIENT's implementation could train only with consecutive integer time points, and so we generated 160 noisy trajectories from the ground-truth SIM350 and SIM690 at $t \in T = \{0,1,2,3,4,5,6,7,8,9\}$. Each of the 1600 simulated vectors in $\{\{\boldsymbol{g}(t)_k \in \mathbb{R}^n\}_{t \in T}\}_{k=1}^{160}$ could be conceptualized as a **single cell**, with `cell_id` $= (t,k)$, at time t with expression values for n genes. We partitioned the cells via the same train-val-test split as the PHOENIX runs, and used this time-labelled "single cell training data" as PRESCIENT's input. PRESCIENT requires a meta data file mapping each `cell_id` to its timepoint $\in T = \{0,1,2,3,4,5,6,7,8,9\}$. We also set the `cell_type` column in the meta-data file to be `regular` for all cells. PRESCIENT models can incorporate proliferation during training via computing a "growth weight" per cell using either lineage tracing data or KEGG gene signatures. In the absence of such data in our simulation, we set this weight to be 1 for all cells. We used the `prescient` package in `Python` to train a model with a single hidden layer of width k_{dim} followed by a softplus activation function. We trained for 2600 epochs and predicted trajectories using PRESCIENT's `net._drift()`

function (that returns $\frac{g(t)}{dt}$) along with `solve_ivp()` from `scipy.integrate`. We optimized k_{dim} based on validation trajectory prediction. We did not find a straightforward functionality in the package to extract a GRN. Because the PRESCIENT implementation was limited to consecutive integer (or equally spaced) time points, this was an impediment to benchmarking PRESCIENT on the yeast and breast cancer datasets.

Additional File 2: Section 3.3.2 Dynamo

Given the input data (simulated gene expression and the corresponding ground truth RNA velocities), Dynamo [3] fits a sparse vector field mapping gene expression to RNA velocity using Gaussian kernel regression. Using the training trajectories from the input data, we created an `vf.SvcVectorField()` object (from the `dynamo` package in `Python`). The trained model (mapping expression to velocity) was used along with `solve_ivp()` from `scipy.integrate` to predict trajectories. Cross-validation was used (based on best MSE in validation trajectory prediction) to choose hyper-parameters M (number of control points) and sparsity parameter λ. MSE was calculated for final model on the test trajectories.

Although `dynamo` provides a straighforward Jacobian function `get_Jacobian()`, the extraction of a GRN using this function has to be done carefully. This is because there is a possibility that the Jacobian is close to zero for both low and high regulator levels; the absolute value of a Jacobian is significantly larger than 0 only within a small range of expression values. Hence we used the following strategy to estimate a GRN:

- We sampled random expression vectors $\boldsymbol{g}_k$ from $\mathcal{N}(0.5, 0.25)$. Since high and low expression values corresponded to 1 and 0 in our simulations, sampling from this Gaussian centered around 0.5 made it less likely that we would be picking very high or low regulator levels.
- To further prevent the Jacobians from being too small, we did sampling in two phases:

  1. We first sampled 5000 random expression vectors $\{\boldsymbol{g}_k \in \mathbb{R}^n\}_{k=1}^{5000}$ from $\mathcal{N}(0.5, 0.25)$, and calculated the 5000 corresponding Jacobian matrices $\{\boldsymbol{J}_k \in \mathbb{R}^{n\times n}\}_{k=1}^{5000}$ using the `get_Jacobian()` function. We computed $\{\mid \boldsymbol{J}_k \mid_{\mathcal{F}} \in \mathbb{R}\}_{k=1}^{5000}$, the Frobenius norms for all the Jacobians. Then we chose $f_{\text{cutoff}} = 95^{\text{th}}$ percentile across the 5000 values in $\{\mid \boldsymbol{J}_k \mid_{\mathcal{F}}\}_{k=1}^{5000}$ as a filtration criterion for the next step.
  2. We sampled a **new batch** of 10000 random expression vectors $\{\boldsymbol{g}_l\}_{l=1}^{10000}$ from $\mathcal{N}(0.5, 0.25)$ and computed $\{\boldsymbol{J}_l\}_{l=1}^{10000}$. We **discarded** any $\boldsymbol{J}_l$ that was “not large enough,” that is if $\mid \boldsymbol{J}_k \mid_{\mathcal{F}} < f_{\text{cutoff}}$, and kept sampling until we had 10000 $\boldsymbol{J}_l$s that satisfied this criterion.

- We used the final list of 10000 Jacobian matrices to compute an average Jacobian $\widetilde{\boldsymbol{E}}$.

Additional File 2: Section 3.3.3 RNA-ODE

Given the input data (simulated gene expression and the corresponding ground truth RNA velocities), RNA-ODE [5] fits black-box random forests mapping expression to velocity. We obtained `Python` source code from `https://github.com/RuishanLiu/VelocytoAnalysis`, to build random forest regressors. We predicted trajectories using the `predict()` function of the random forest along with `solve_ivp()` from `scipy.integrate`. We optimized the number of trees in the forest (`n_estimators`) based on validation trajectory prediction. The source code also has a `GET_GRN()` function to easily estimate a $\widetilde{\boldsymbol{E}}$ based on the fitted model using a GENIE3-like approach.

Additional File 2: Section 3.3.4 DeepVelo

Given the input data (simulated gene expression and the corresponding ground truth RNA velocities), DeepVelo [12] fits a black-box autoencoder mapping expression to velocity. We obtained `Python` source code from `https://github.com/gersteinlab/DeepVelo`, to build a 4 layer encoder and 4 layer decoder with ℓ_1 regularization. We used size $4p$ for the intermediate layers and size p for the latent layers. We optimized p (and hence the number of neurons in each layer of the autoencoder) based on validation trajectory prediction. Further details can be found on our GitHub repository [37]. We predicted trajectories using the auto-encoder's `predict()` function along with `solve_ivp()` from `scipy.integrate`. The source code also has functionalities to estimate $\widetilde{\boldsymbol{E}}$ a gene-correlation matrix of cells, based on simulating "retrograde trajectories."

Additional File 2: Section 4 Creating *in silico* data

Additional File 2: Section 4.1 Ground truth system using SimulatorGRN

We created a ground truth gene regulatory network (GRN) by sampling from S. cerevisiae (yeast) regulatory networks obtained from the SynTReN v1.2 supplementary data in simple interaction format (SIF) [71]. The SynTReN file provides a directional GRN containing 690 genes and 1094 edges with annotations (activating vs repressive) for edge types; we defined this GRN to be ground truth network G_{690}. To obtain G_{350}, we used `SimulatorGRN` [35] in `R` to sample a subnetwork of 350 genes and 590 edges from G_{690}, using the `sampleGraph()` function. Next, to each edge (for instance from gene A to gene B), we assigned randomly generated $EC_{50}^{AB} \in (0.4, 0.6)$ and $\eta^{AB} \in (1.39, 1.8)$ values using the `randomizePrams()` function from `SimulatorGRN`; lists of edges with EC_{50}^{AB}, η^{AB} values are provided with the PHOENIX release [37]. Each EC_{50}^{AB}, η^{AB} pair defines the relationship between a regulator A and its target B. The `simulationGRN()` function then used the edges in G_{350} and G_{690} to define systems (SIM350 and SIM690) of normalised-Hill ODEs [29], where the activation of B by a single regulator A was modelled as:
$\frac{dB}{dt} = f_{act}(A, EC_{50}^{AB}, \eta^{AB}) - B = \frac{\beta A^{\eta^{AB}}}{\beta - 1 + A^{\eta^{AB}}} - B$, where $\beta = \frac{EC_{50}^{AB} - 1}{2EC_{50}^{AB} - 1}$. Repression of B by A was simply modelled using $1 - f_{act}(A, EC_{50}^{AB}, \eta^{AB})$. Co-regulation by A_1 and A_2 was modelled as either a logical `AND`:

$$f_{act}(A_1, EC_{50}^{A_1B}, \eta^{A_1B}) \times f_{act}(A_2, EC_{50}^{A_2B}, \eta^{A_2B}),$$

or a logical `OR` gate:

$$\begin{aligned} &f_{act}(A_1, EC_{50}^{A_1B}, \eta^{A_1B}) + f_{act}(A_2, EC_{50}^{A_2B}, \eta^{A_2B}) \\ &- f_{act}(A_1, EC_{50}^{A_1B}, \eta^{A_1B}) \times f_{act}(A_2, EC_{50}^{A_2B}, \eta^{A_2B}) \end{aligned}$$

where we randomly assigned 90% of co-regulations as logical `AND`s, and the other 10% as logical `OR`s. Proceeding in this manner, `simulationGRN()` used the network structures to define dynamical systems SIM350 and SIM690, containing an ODE for each gene in each of G_{350} and G_{690} respectively. For any gene Z that has no upstream regulators, we assign $dZ/dt = 0$. We simulated time-series expression from these dynamical systems using `deSolve` in R.

Additional File 2: Section 4.2 Creating corrupted/misspecified and incomplete prior models

Corrupted prior:
For each noise level $\sigma_{\%} \in \{0\%, 5\%, 10\%, 20\%, 40\%, 80\%, 100\%\}$ in our *in silico* experiments, we created a shuffled version of G_{350} (and similarly G_{690}) where we shuffled $\sigma_{\%}$ of the edges by relocating those edges to new randomly chosen origin and destination genes within the network. This yielded the shuffled network $G_{350}^{\sigma\%}$ (and similarly $G_{690}^{\sigma\%}$) with corresponding adjacency matrix $\boldsymbol{A}^{\boldsymbol{\sigma\%}}$. We set activating edges in $\boldsymbol{A}^{\boldsymbol{\sigma\%}}$ to $+1$ and repressive edges to -1, and defined the simple linear prior domain knowledge model: $\mathcal{P}^*\left(\boldsymbol{\gamma_k}\right) = \boldsymbol{A}^{\boldsymbol{\sigma\%}} \cdot \boldsymbol{\gamma_k} - \boldsymbol{\gamma_k}$.

Incomplete prior:
For each withhold percentage $w_{\%} \in \{5\%, 10\%, 25\%, 50\%, 75\%, 100\%\}$, we repeated the same procedure as above, with a key modification. Instead of randomly relocating $w_{\%}$ of the edges of G_{350} (and similarly G_{690}), we completely removed those edges from the network, by setting the corresponding entries in the adjacency matrix $\boldsymbol{A}^{\boldsymbol{w\%}}$ to be 0. Similarly as above, we then defined the prior domain knowledge model: $\mathcal{P}^*\left(\boldsymbol{\gamma_k}\right) = \boldsymbol{A}^{\boldsymbol{w\%}} \cdot \boldsymbol{\gamma_k} - \boldsymbol{\gamma_k}$.
