# Supplementary material for: Biologically informed NeuralODEs for genome-wide regulatory dynamics
Source: Genome Biol. 2024 May 21;25:127. doi: 10.1186/s13059-024-03264-0 (PMC11106922; doi:10.1186/s13059-024-03264-0)
Supplement: Supplementary file 3 — Additional file 3: Peer review history. [file 13059_2024_3264_MOESM3_ESM.docx]

**Review history**

**First round of review**

**Reviewer 1**

In this article, the authors proposed a new NeuralODE-based method to better facilitate modeling of gene regulatory networks by combining the flexibility of ODE models and the prior system biology knowledge.
Major Comments
In the introduction section, the authors argued that one of the drawbacks of the existing models for GRN inference was the reliance of dimension reduction techniques as a preprocessing step. However, there have been countless evidence in both computational biology and machine learning in general showing that feature selection does benefit a lot in model construction and training by reducing the signal-to-noise ratio and reducing the number of parameters needed to train the model, effectively reducing the probability of overfitting, whereas the authors claimed in the manuscript that PHOENIX just used the original expression matrix as the input. The authors might consider explain more clearly the reasoning behind the choice of abandoning feature selections and provide more clear evidence showing that training without feature selection works better than training with feature selection.
In page 12, the authors discussed and compared between various existing methods of GRN inference, also shown in Table 1. These comparisons were more theoretical by nature and the authors did not provide a lot of result-based evidence. The authors might consider moving these comparisons to a more appropriate space in the manuscript, such as the introduction or the methods sections.
From Figure 4, bottom-right, it seemed like that the performance of PHONEIX heavily depended on the existence and the amount of used-supplied prior knowledge. Either too much or too little prior knowledge would significantly decrease its performance. However, in the manuscript, the authors did not cover a lot on this topic (line 616-626); nor did the authors add any direct comparisons with other existing models on the real dataset. The authors might consider adding more explanations and comparisons to better assess and demonstrate the performance of PHONEIX over other existing models.
In Figure 5, the authors also only displayed the performance of PHONEIX alone with a list of pathways. It would be better to show more comparisons between other models in terms of performance and a more concise and meaningful list of pathways with accession IDs available for reproducibility.
It seemed that PHONEIX heavily depends on the "used-supplied prior knowledge". In the text describing the equations to calculate the prior knowledge, the authors used the term "ghost input" γ and "the adjacency matrix A of likely network structure based on prior domain knowledge" without clear definitions. The authors might want to clarify more on the exact deriviation and calculation involving these two inputs.

Minor Comments
In the introduction section, the authors briefly mentioned the drawbacks of the two types of existing models for GRN inference. However, the issues with each type of models were not addressed very clearly, especially in the third paragraph at page 3 (line 117-119). The authors might consider refine the wording such that the issues are indicated more directly.
The authors might consider adding sub-panel identifiers to the figures. It will be much more concise to refer to a figure using the identifiers than using words such as "Figure 4, bottom-right".

**Reviewer 2**

Hossain et al present a new model for gene regulation, based around neural ODEs. The concept is that a neural net learns a mapping from gene expression X to rate of change dX/dt, and then an ODE solver estimates the gene expression at future times. They constrain their model to encode biological connections using a modified loss function that incorporates known, prior information about TF to gene binding, so that model coefficients which do not have TF binding support are regularized more strongly. Finally, they extract a gene regulatory network by ranking neural network model coefficients. This is a well-written manuscript with a comprehensive review of existing literature in the field, and well-chosen comparison methods.

I love this model conceptually. Neural models motivated by the kinetic equations that actually represent the biological system are a fantastic blend between classic biochemistry and modern machine learning. If you asked me the best way forward for the network inference field, I would show you this manuscript.

I did have a lot of trouble telling from this manuscript if the model the authors built has actually worked. The following concerns should be reasonable to address.

1. The argument for including learned gene-specific scaling parameter v is very questionable. Why would this not be captured in the model coefficients? Because the model coefficients used for GRN reconstruction need to be scaled? A clear explanation of what this parameter is intended to do is essential, and my recommendation is to reformulate this model without it.
2. Is this framework amenable to increasing the neural network complexity with more layers? Please add to the discussion.
3. The prior sign is randomly assigned to +1/-1? Please explain this choice, as it appears very odd.
4. The simulated test does an excellent job showing that this method might work. However, even in this simulated test, it appears that the gold standard used for GRN scoring was embedded in the model via the loss prior. It is essential that either this method is tested on GRN information that is held out of the prior, or the method is described as pruning a known network.
5. The "high noise" tests in the simulated data are unlikely to reflect the magnitude of biological noise in real-world data. I would expect results for Figure 3 and Supp Table 1 to be reported for a full 0-100% noise range for both prior and expression data.
6. It's not clear to me which "transition pairs" were used for validation or test, and it's not clear to me if both times validation & test transition pairs are held out of the training data. Confidence in the test metrics reported is diminished greatly if pair (2, 3) is test data, but (1, 2) and (2, 3) are training. This must be clarified.
7. It's also not clear why only adjacent transition pairs are used here instead of using longer trajectories. This choice should be explicitly justified.
8. I was not able to determine how the "normalized gene expression" score was generated for Figure 4, or what it actually means. I am concerned that the range of expression values is inconsistent with what I would expect for these genes. A clearer description of this preprocessing is necessary. Please also report results for ACT1 and TDH1.
9. The trained yeast cell cycle model must be applied to a data set independent of the training data, and the comparative results must be reported. Fortunately, GSE3635 from the same authors, using the same platform, appears to be perfect for this.
10. To be perfectly honest, I do not know what I am supposed to take away from the breast cancer analysis. The pseudotime ordering for different patients is questionable at best, and the analyses are concerning. I would not expect frozen clinical samples to even be correlated to 0.99, much less to be predictive at 0.99, and so I am concerned that the preprocessing has removed much of the biological signal. Additional supplemental figures showing gene expression over time and comparable predictions in this data set are essential.
11. I am very concerned that the authors have not tested the modeling against RNA-seq data. The microarray data used is two-channel for yeast, and it's not clear if this means that the RNA velocity estimated for the yeast data is actually rate of change of fold change. The affymetrix array used for the breast cancer data has its own problems with linear response. It is necessary to add an RNA-seq (or single cell RNA-seq) trained model of the authors' choice to this manuscript. Please choose one with training data that has n > 2.

**Reviewer 3**

this manuscript, Hossain et al. presented a PHOENIX framework, which uses neural ODEs to derive biologically interpretable ODE models for gene regulatory networks from scRNA-seq data. Learning dynamical models from high throughput single cell data has become an active research area in recent years. The authors nicely summarized some recent efforts. Their PHOENIX framework aims to tackle two important issues: how to construct a biologically interpretable model, and how to incorporate prior knowledges in the learning process. The authors used the NeuralODE architecture to approximate the vector field functions of an ODE model , and tested on two in silico datasets, as well as a breast cancer progression dataset. I am interested in seeing such timely and highly significant efforts, while I have some major concerns that need the authors to address:

1) The authors benchmarked their method against several other existing methods using in silico data, and concluded their method has the best performance. I am afraid that the comparison is unfair. The authors confused the applicability of several methods. While they nicely classed the existing methods into two groups, "one-step" and "two-step" methods, for those methods, a more fundamental difference is whether one uses time-series single cell data or snapshot single cell data. Careful reading on the supplemental documents reveals that the authors used time series single cell data as input for methods like PHOENIX and RNAForecaster. It is not surprising at all that they outperformed those "two-step" methods, as reliable causal inference requires true time series data.
2) Notice in practice it is challenging (actually impossible at the moment) to record high dimensional single cell expression state over time. The authors suggest to use pseudotime trajectories, which are inferred from snapshot data. Then it comes back to how to generate reliable pseudotime trajectories. In principle with additional information (such as RNA velocities, provided they are reliable), one can have better inference on the pseudotime (or real time) trajectories. In that sense, one may argue that PHOENIX provides a general approach to infer interpretable GRNs and ODE models from outputs of other methods (e.g. Dynamo), which are currently less transparent.
3) Related to point 1, for those snapshot-based methods, it is not appropriate for them to generate the in silico data from a single set of trajectories with recorded cell states of each cell at different time points. Instead, they should randomly (or based on certain initial condition) select 160 initial cell states as t0, randomly select another 160 initial cell states and propagate to time t1, and so on, which closely mimic an experimental setup.
4) For the two yeast models, how many parameters need to be determined? Specifically, how to decide on the number of regulators acting on a given gene? How to balance between fitting accuracy and the risk of overfitting?
5) For the breast cancer data, I am surprised that with one single pseudotime trajectory they can recover such a complicated model. I would like to see more technical details to be convinced that there is no overfitting, and the results are conclusive—that is, one can unambiguously distinguish different competing models.
6) I suspect that the accuracy of the methods, esp. the "two-step" approaches have some minimum requirements on the sampling size. The authors may examine systematically how the performances of these methods change when they change the number of sampled cells.
7) A few comments on the methods they tested:
a) Supp. Methods 3.3.1: PRESCIENT uses time courses of snapshot distributions, and assume that one can not follow individual cells over time. PRESCIENT assumes a scalar potential governs the systems dynamics. This is generally a questionable assumption for thermodynamic nonequilibrium system (as a cell is), especially when cell cycle is explicitly considered.
b) I read the preprint of RNAForecaster (https://www.biorxiv.org/content/10.1101/2022.08.04.502825v2.full). It seems that these authors are also confused about the metabolic labeling method, and assumed that one can measure the mRNA counts of the SAME cell at two time points (Fig. 1 in that preprint). This is incorrect. The experimental methods such as scEU-seq are destructive, so one CANNOT track one cell at two different time points.
c) Supp. Methods 3.3.2 Dynamo: The authors use the average Jacobian to extract a GRN. While this is a method used in the original Dynamo paper, there is a caveat with this method. Notice that for a sigmoidal shaped dose-response function with high nonlinearity (i.e., large Hill coefficient), the Jacobian is close to zero for both low and high regulator levels----in the case with a very large Hill coefficient, the absolute value of a Jacobian is significantly larger than 0 only within a small range centered around K_M; the average Jacobian can be very small if this transition region is not well sampled. The summary in Table 1 is inaccurate. Dynamo is not restricted to metabolic labeling data, fewer than 300 genes, and top 30 PC/2D UMAP---the authors might get this wrong impression from some specific examples in that paper.

**Authors’ response to reviewers**

We thank all reviewers for the positive assessment of our work and the highly constructive feedback. In response, we have made a number of substantive changes that we hope you agree has produced an even better manuscript.

# Response to Reviewer 1

**Reviewer Comment 1.1** — In the introduction section, the authors argued that one of the drawbacks of the existing models for GRN inference was the reliance of dimension reduction tech- niques as a preprocessing step. However, there have been countless evidence in both computational biology and machine learning in general showing that feature selection does benefit a lot in model construction and training by reducing the signal-to-noise ratio and reducing the number of pa- rameters needed to train the model, effectively reducing the probability of overfitting, whereas the authors claimed in the manuscript that PHOENIX just used the original expression matrix as the input. The authors might consider explain more clearly the reasoning behind the choice of aban- doning feature selections and provide more clear evidence showing that training without feature selection works better than training with feature selection.

**Reply**: We agree that, in general, dimension reduction and feature selection have been of use in some areas of computational biology. For this particular application of inferring gene regulatory dynamics, dimensionality reduction—used by several methods—hinders interpretability, as the learned model op- erates on this latent space rather than the original gene space. It is further not (trivially) possible to map back to the full gene space to infer a GRN. Feature selection as a preprocessing step sacrifices important relations involving the unselected genes; we show that in breast cancer considering the full set of genes provides much more biological meaningful insights than considering for example the top-k most varying genes, as common in the literature. A built-in (target aware) feature selection would indeed help in reducing the number of parameters but existing methods however do not make use of that. In fact, the prior regularization of PHX acts as implicit feature selection method by effectively driving those unimportant relations close to zero. This feature selection is however on the edge (gene-gene interaction) rather than node (gene) level, which allows us to still recover dynamics that involve genes that are targeted in highly specific ways and hence might not exhibit such strong variance. We adapted the language in the manuscript to better reflect these points.

**Reviewer Comment 1.2** — In page 12, the authors discussed and compared between various existing methods of GRN inference, also shown in Table 1. These comparisons were more theoretical by nature and the authors did not provide a lot of result-based evidence. The authors might consider moving these comparisons to a more appropriate space in the manuscript, such as the introduction or the methods sections.

**Reply**: We appreciate the suggestion and have moved the table to the supplement. Instead, we present additional quantitative evaluation of the methods on real data in new **Table 1**, **Figure 6**, and **Supp. Table 11** to highlight the main advantages of our approach.

**Reviewer Comment 1.3** — From Figure 4, bottom-right, it seemed like that the performance of PHONEIX heavily depended on the existence and the amount of used-supplied prior knowledge. Either too much or too little prior knowledge would significantly decrease its performance. How- ever, in the manuscript, the authors did not cover a lot on this topic (line 616-626); nor did the authors add any direct comparisons with other existing models on the real dataset. The authors might consider adding more explanations and comparisons to better assess and demonstrate the performance of PHONEIX over other existing models.

**Reply**: PHOENIX, by design as well as in contrast to other methods, does indeed rely on the soft constraints derived from the provided prior knowledge. The implicit constraints introduced in this way allow the model to learn a sparse, biologically meaningful network representation of gene regulatory dynamics, which otherwise would not be possible. To further demonstrate this point, we added a comparison with competing methods on both yeast and breast cancer (see new **Table 1**, **Figure 6**, and **Supp. Table 11**), that show that other approaches, which do not harness prior knowledge, perform much worse than PHOENIX in terms of learning the underlying GRN. We extended the discussion on this topic accordingly and note that the (prior based) regularization should be tuned for each data, which in practice we do with a validation set.

**Reviewer Comment 1.4** — In Figure 5, the authors also only displayed the performance of PHONEIX alone with a list of pathways. It would be better to show more comparisons between other models in terms of performance and a more concise and meaningful list of pathways with accession IDs available for reproducibility.

**Reply**: We have updated the corresponding section to present a more quantitative comparison of various methods on the breast cancer data (**Supp. Table 11** and **Figure 6**), which further highlights the benefits of PHOENIX in terms of scalability and interpretability.

With the analysis of **Figure 5** we try to convey that the larger models that are obtainable with PHOENIX capture a finer grained and more comprehensive picture of the biological pathways that are involved in the network–which is also reflected in the stark difference in explanatory performance comparing PHOENIX with existing methods. We are convinced that our exploratory analysis on the gene and pathway influence scores provides evidence of the limitations of the smaller models. Also, we have now created **Supp. Table 9** with all the relevant reactome accession IDs for reproducibility.

**Reviewer Comment 1.5** — It seemed that PHONEIX heavily depends on the “used-supplied prior knowledge”. In the text describing the equations to calculate the prior knowledge, the authors used the term “ghost input” *γ* and “the adjacency matrix *A* of likely network structure based on prior domain knowledge” without clear definitions. The authors might want to clarify more on the exact deriviation and calculation involving these two inputs.

**Reply**: As clarified in Reviewer Comment 1.3 PHOENIX does rely on the provided prior knowledge by construction. The biologically informed constraints introduced in this way is what allows PHOENIX to be highly performant while still being explainable. The constraints we introduce are simply a way of ac- knowledging the fact that transcription factors have specific binding motifs and, as such, are most likely to regulate genes with those sites in regulatory regions—something that guides the predicted dynamics.

To address your comment, we provide additional clarification on ***γ*** and ***A*** as follows:

- ***A*** is the adjacency matrix of likely network structure based on prior domain knowledge. Here, we usually use transcription factor binding motif occurrences in promoter regions of target genes as a basis for ***A***, where an entry *A_ij_* = 1 encodes that TF *i* likely binds to the promoter of gene *j*. We provide details on how these motif based prior networks encoded by ***A*** are constructed for each analysis in **Methods 1.3**, **2.2**, **3.2**, and **4.2**. We further added clarifications for the motivation of this prior network in the introduction:

“*In the optimization step of PHOENIX, we introduce user-defined prior knowledge in the form of a ‘network prior.’ Here, we derive a prior based on TF binding motif enrichment that is predicated on the understanding that the direct regulators of gene expression are transcription factors that bind in the region of a gene’s transcription start site (TSS). Because most transcription factors bind to distinct sequence motifs, each transcription factor has the potential to regulate only a fixed subset of genes. This regulatory constraint can be expressed as a network prior by mapping transcription factors to the promoter sequence of regulated genes, using tools such as FIMO and GenomicRanges.*”.

- ***γ***(*t_i_*)s are simulated expression values that are used to link the prior model to the neuralODE por- tion of PHOENIX. This is described in the section “Structural domain knowledge incorporation,” and further details about the operationalization of the ***γ***(*t_i_*)s can be found in **Supp. Methods**

**1.2**. Because these vectors are **simulated** expression values, we also refer to them as “ghost” expression inputs/values. We have added the following sentence to the relevant text and figure captions to make this clear for the reader:

*“Since the* ***γ***(*t_i_*)*s were simulated expression values, we also refer to them as ‘ghost inputs.’ More details about their operationalization can be found in Supp. Methods 1.2.”*

**Reviewer Comment 1.6** — In the introduction section, the authors briefly mentioned the drawbacks of the two types of existing models for GRN inference. However, the issues with each type of models were not addressed very clearly, especially in the third paragraph at page 3 (line 117-119). The authors might consider refine the wording such that the issues are indicated more directly.

**Reply**: We have modified the text of that paragraph to better describe the limitations of existing methods and to better distinguish the advances represented in PHOENIX. These are also summarized in **Supp. Table 7** to provide an overview. Furthermore, **Supp. Table 3**, **Supp. Table 4**, and **Supp. Table 5** use the simulated data to highlight PHOENIX’s great advantage in learning biologically meaningful gene regulatory dynamics.

**Reviewer Comment 1.7** — The authors might consider adding sub-panel identifiers to the figures. It will be much more concise to refer to a figure using the identifiers than using words such as ”Figure 4, bottom-right”.

**Reply**: We have modified all multi-panel figures, as well as their corresponding captions and references, to include subpanel-identifiers.

# Response to Reviewer 2

**Reviewer Comment 2.1** — The argument for including learned gene-specific scaling parameter *ν* is very questionable. Why would this not be captured in the model coefficients? Because the model coefficients used for GRN reconstruction need to be scaled? A clear explanation of what this parameter is intended to do is essential, and my recommendation is to reformulate this model without it.

**Reply**: Indeed, appropriate scaling of the model coefficients would account for *υ* and, therefore, *υ* would be integrated in the final model parameters. However, a formulation with separate *υ* induces technical advantages. Having the scaling *υ* as a separate learnable parameter helps in robustly opti- mizing for a good model. In particular, ODEs have difficulties with learning zero gradients and this technical choice of modeling *υ* separately helps to explicitly learn these zero gradients correctly. We added this clarification to the manuscript along with new results in Section **Supp. Fig. 5** that shows the difference of a fitted zero gradient between PHOENIX with and without scaling *υ*.

Regarding the evaluation of the encoded GRN, the model coefficients are appropriately scaled to account for *υ* (see **Supp. Methods 2**).

**Reviewer Comment 2.2** — Is this framework amenable to increasing the neural network com- plexity with more layers? Please add to the discussion.

**Reply**: Thank you for the interesting suggestion. We had considered this but were hesitant to specu- late too much in the paper. With your encouragement, we have added the following to the end of the discussion:

*Although PHOENIX, in its current implementation, is designed with one “layer” of regulation to model TF-gene interactions, we recognize that there are other regulatory elements or higher order regulatory effects in the cell that contribute to the control of gene expression. Such additional effects could po- tentially be modeled by increasing the complexity of the NeuralODE solver by introducing additional layers to the NeuralODE framework. However, this would increase the computational requirements by PHOENIX and reduce its current advantage of being a light-weight method.*

**Reviewer Comment 2.3** — The prior sign is randomly assigned to +1/-1? Please explain this choice, as it appears very odd.

**Reply**: Originally, we designed this method to reflect priors with sign information (representing either repressive or activating behaviour). For many real-world applications, including the ones considered here, such a sign information is often not available and we decided to introduce signs such that on expectation the assigned sign is zero—meaning that on expectation we do the least error globally. After your thoughtful comment we revisited this formulation, in particular the randomness introduced by this procedure and what happens to an individual edge in the GRN. We have now run additional experiments that include assigning + signs to everything in the prior. On synthetic data, we show that despite the loss of sign-information, this barely harms performance (see new **Supp. Table 2**). We further reran all experiments on real data with this deterministic sign assignment that better reflects the biological

meaning of the given prior and **report this as results in the new draft**. This modification actually leads to much more accurate GRNs on the real datasets. Note that PHOENIX can still recover true negative edges with an all-positive prior.

**Reviewer Comment 2.4** — The simulated test does an excellent job showing that this method might work. However, even in this simulated test, it appears that the gold standard used for GRN scoring was embedded in the model via the loss prior. It is essential that either this method is tested on GRN information that is held out of the prior, or the method is described as pruning a known network.

**Reply**: As described in the results section (snippet included here) our simulation experiments did encompass different levels of noise being applied to the prior (a corrupted prior) that was provided to PHOENIX:

*“Since the inclusion of such static prior knowledge greatly increased the explainability of the inferred dynamics, we also investigated how explainability was affected by misspecification of the prior. In our in silico experiments, we had randomly corrupted (misspecified) the prior by an amount commensurate with the noise level.”*

So, in all our simulation experiments, a noise level of *p*% not only reflects the amount of Gaussian noise added to expression values, but also corresponds to a corrupted prior where *p*% of the edges were deleted and replaced with new (wrong) edges (details of this corruption procedure are provided in **Supp. Methods 4.2**). We observed that even when provided corrupted prior information, PHOENIX was able to recapitulate the gold-standard network with higher accuracy than that of the corrupted prior used to train it. (**Figure 3** and **Supp. Table 1**).

Furthermore, as discussed above in Reviewer Comment 2.3 we now provide additional results in **Supp. Table 2**, where no sign information about prior edges is given to PHOENIX (so all prior edges are forced to have a positive sign), modeling a standard scenario for real-world data, where information of whether an interaction is activating or repressing is not known. Even in this more challenging scenario, PHOENIX’s performance is comparable to when perfect sign information was provided in the prior.

**Reviewer Comment 2.5** — The “high noise” tests in the simulated data are unlikely to reflect the magnitude of biological noise in real-world data. I would expect results for Figure 3 and Supp Table 1 to be reported for a full 0-100% noise range for both prior and expression data.

**Reply**: As discussed in Reviewer Comment 2.4 this noise is applied to **both** the prior and the expression data, which we think does model real biological noise considering 10% or 20% of noise. We agree, however, that the labeling of *high* noise might be misleading and changed this accordingly. As requested, we further extended our simulation experiments to three additional noise settings (40%, 80% and 100%). We have updated all relevant tables (**Supp. Table 1**; **Supp. Table 3**; **Supp. Table 4**; **Supp. Table 5**) and figures (**Supp. Fig. 2**; **Supp. Fig. 4**,) to include these new noise settings, and have generated additional supplementary figures (**Supp. Fig. 1**; **Supp. Fig. 3**) where necessary.

**Reviewer Comment 2.6** — It’s not clear to me which “transition pairs” were used for validation or test, and it’s not clear to me if both times validation & test transition pairs are held out of the training data. Confidence in the test metrics reported is diminished greatly if pair (2, 3) is test data, but (1, 2) and (2, 3) are training. This must be clarified.

**Reviewer Comment 2.7** — It’s also not clear why only adjacent transition pairs are used here instead of using longer trajectories. This choice should be explicitly justified.

**Reviewer Comment 2.8** — The trained yeast cell cycle model must be applied to a data set independent of the training data, and the comparative results must be reported. Fortunately, GSE3635 from the same authors, using the same platform, appears to be perfect for this.

**Reply**: **(to 2.6, 2.7, and 2.8)** We adapted all of your suggestions and they indeed provide a more compelling evidence that PHOENIX can accurately learn gene regulatory dynamics. We also removed the misleading terminology of ”transition pairs”. In more detail, we now used one of the two yeast replicates for training (train + validation splits on this replicate) and the other replicate for testing (not seen in any way during training). Furthermore, within the replicate for training, we used longer contiguous sections of the trajectory for training and validation, ensuring that the train and validation sets are disjunct. We provide a description of these new experiments in the updated draft (see **Methods 2.2**). All reported yeast results (including those visualized in **Figure 4**) are under this new training and testing scheme. Also, all reported new results are using the suggested change (in Reviewer Comment 2.3) to the prior edges (all edges have positive sign). Note the apparent advantage of PHOENIX over competing methods (**Table 1**).

**Reviewer Comment 2.9** — I was not able to determine how the “normalized gene expression” score was generated for Figure 4, or what it actually means. I am concerned that the range of expression values is inconsistent with what I would expect for these genes. A clearer description of this preprocessing is necessary. Please also report results for ACT1 and TDH1.

**Reply**: Before, we did normalize this gene expression to [0,1] through a simple min-max normalization for visualization. We agree that this might hinder interpretability for someone interested in the actual expression values. We have now reverted to reporting the original microarray expression values (the usual fold-changes) in the updated **Figure 4**. We believe that the undoing of the re-normalizing of gene expression values for visualization should address the concerns of the referee appropriately. All preprocessing details can be found in **Methods 2.1**.

As requested, we have plotted the observed and predicted trajectories of ACT1 and TDH1 in **Supp. Fig. 6**. Both ACT1 and TDH1 show very low expression and expression changes within the experiment compared to other genes (for comparison, we put them on the same axis scale as the original examples in the main paper), which makes a statement about how well PHOENIX fitted these genes as there is little complexity to model.

**Reviewer Comment 2.10** — To be perfectly honest, I do not know what I am supposed to take away from the breast cancer analysis. The pseudotime ordering for different patients is questionable at best, and the analyses are concerning. I would not expect frozen clinical samples to even be correlated to 0.99, much less to be predictive at 0.99, and so I am concerned that the preprocessing has removed much of the biological signal. Additional supplemental figures showing gene expression over time and comparable predictions in this data set are essential.

**Reply**: The reported *R*^2^ values were indeed very high given such difficult data. After careful investi- gation into the matter, we found that it is neither a surprisingly high quality, nor a preprocessing issue that removed biological signal. Instead, the high *R*^2^ is largely driven by most genes showing no dynamic expression, which are hence easily estimated by a constant trajectory. Considering only the performance on the top most variable genes, we observe a significant decrease in *R*^2^. A second driver of the high *R*^2^ is that estimating a future gene expression value based on the immediate previous time point seems to oversimplify the task. We addressed these issues by:

1. adding a careful discussion on the impact of static gene expression on *R*^2^, along with a supple- mentary plot **Supp. Fig. 10** that shows how the change in performance when considering only the top most varying genes in the evaluation.
2. changing the evaluation strategy estimating a future timepoint based on only the initially observed gene expression value (*t* = *t*_0_) in the test set.

**Reviewer Comment 2.11** — I am very concerned that the authors have not tested the modeling against RNA-seq data. The microarray data used is two-channel for yeast, and it’s not clear if this means that the RNA velocity estimated for the yeast data is actually rate of change of fold change. The affymetrix array used for the breast cancer data has its own problems with linear response. It is necessary to add an RNA-seq (or single cell RNA-seq) trained model of the authors’ choice to this manuscript. Please choose one with training data that has *n >* 2.

**Reply**: We added an additional small experiment of RNA-seq timecourse data of six timepoints of primary B-cells, where one group of cells has been treated with Rituximab and another group has been grown without treatment, two replicates each. To complement the breast cancer analysis, in this proof of concept we focused on a differential analysis comparing two PHOENIX models between the two con- ditions (treated vs untreated). We could validate that many of the regulators with the largest difference in regulatory effect between the two conditions are associated with Apoptosis, the primary mechanism of action of Rituximab (**Supp. Fig. 11**). Given the scope of this Methods article we did not push for a further exploratory analysis.

Unfortunately, it is very hard to come by an actual timecourse dataset with *n >>* 2, let alone one of good quality. For learning, what is more important is the number of timepoints, as these determine the (technical) number of samples we have for optimization. We do appreciate the concern and think that for more heterogeneous data sources, such as a tumor, models trained with small *n* (number of patients) might recover patient (background) specific effects in their trajectories, rather than common tumor-related processes. We are looking forward to the exciting current experimental efforts to gather larger longitudinal data.

# Response to Reviewer 3

**Reviewer Comment 3.1** — The authors benchmarked their method against several other exist- ing methods using in silico data, and concluded their method has the best performance. I am afraid that the comparison is unfair. The authors confused the applicability of several methods. While they nicely classed the existing methods into two groups, “one-step” and “two-step” methods, for those methods, a more fundamental difference is whether one uses time-series single cell data or snapshot single cell data. Careful reading on the supplemental documents reveals that the authors used time series single cell data as input for methods like PHOENIX and RNAForecaster. It is not surprising at all that they outperformed those “two-step” methods, as reliable causal inference requires true time series data.

**Reply**: (Un)fortunately, there are only very few methods that are capable of predicting actual gene expression dynamics. Yet, it has to be shown that the achieved performance with a novel method can not be trivially reached by adapting an established method to the new data type. We agree that this is an apples to oranges comparison, but for the aforementioned reason one that has to be done. We carefully revised the language to make the difference between the methods clearer and highlight that, while worse in performance on this time-course data, these methods can be applied to snapshot data, which the one-step approaches cannot.

*“A second class of approaches consists of “two-step” methods such as Dynamo, RNA-ODE, and Deep- Velo that instead only require snapshot expression data learning f with two separate steps, which allows for much broader applicability to standard RNA-seq data at the cost of performance, as true timecourse data is required for reliable causal inference.”*

Furthermore, we want to note that in the simulation study we provided the snapshot approaches with the **ground truth** velocity from the data generating process to make up for the possible disadvantage, which is information that none of the one-shot approaches was given.

**Reviewer Comment 3.2** — Notice in practice it is challenging (actually impossible at the moment) to record high dimensional single cell expression state over time. The authors suggest to use pseudotime trajectories, which are inferred from snapshot data. Then it comes back to how to generate reliable pseudotime trajectories. In principle with additional information (such as RNA velocities, provided they are reliable), one can have better inference on the pseudotime (or real time) trajectories. In that sense, one may argue that PHOENIX provides a general approach to infer interpretable GRNs and ODE models from outputs of other methods (e.g. Dynamo), which are currently less transparent.

**Reply**: This is a great suggestion. In the discussion, we have “borrowed” some of your language to admit this possibility:

*“One of the challenges in modeling the evolution of network processes, of course, is obtaining data sets for which temporal data are available. However, we recognize that in any data set, the individual samples represent a continuum of states between health and disease and so can use pseudotemporal*

*ordered samples. But this too has some limitations as there is as of yet no established method for order- ing bulk samples–although there have been some methods for single-cell data adapted to “bulk” tissue samples. This admits an interesting possibility: one could use additional information (such as RNA ve- locities, provided they are reliable) from which one could infer pseudotime (or real-time) trajectories. In that sense, one may argue that PHOENIX provides a general approach to infer interpretable GRNs and ODE models from outputs of other methods (such as Dynamo), which are currently less transparent. This could provide the best of both worlds with a reduced dimensional approach to temporal ordering providing input for a more complete and interpretable final model.”*

**Reviewer Comment 3.3** — Related to Reviewer Comment 3.1, for those snapshot-based meth- ods, it is not appropriate for them to generate the in silico data from a single set of trajectories with recorded cell states of each cell at different time points. Instead, they should randomly (or based on certain initial condition) select 160 initial cell states as *t*_0_, randomly select another 160 initial cell states and propagate to time *t*_1_, and so on, which closely mimic an experimental setup. I suspect that the accuracy of the methods, esp. the “two-step” approaches have some minimum requirements on the sampling size. The authors may examine systematically how the performances of these methods change when they change the number of sampled cells.

**Reply**: We appreciate that snapshot-based methods have been designed for a different use case, namely a data generating process that would be closer to the synthetic data experiments suggested by the reviewer. Their performance might indeed depend on the number of sampled cells/trajectories.

However, our work focuses on modeling time-series expression data, for which the analyzed synthetic data generating process is representative. Note that any modeling attempt for this scenario, while being highly relevant, is quite novel. Therefore, there not many modeling approaches have been proposed that we could compare with. The considered snapshot-based approaches come closest to our intended use cases, in particular, if they are NeuralODE based.

We found it reasonable that one might be able to transfer variations of snapshot-based models to our one-step scenario. In the revised manuscript, we clarify the reasons for our comparisons and highlight the different intentions in model design. In addition, to make the comparison more fair and level the playing field, we have provided **ground truth** velocities to two-step methods. Our results suggest that PHOENIX is better suited for our intended application scenario than two-shot approaches even under idealized conditions.

**Reviewer Comment 3.4** — For the two yeast models, how many parameters need to be deter- mined? Specifically, how to decide on the number of regulators acting on a given gene? How to balance between fitting accuracy and the risk of overfitting?

**Reply**: For the two yeast models (PHX with prior and PHX without prior), the number of parameters is the same. The number of parameters is determined by the number of genes (*n*) and the number of hidden neurons (*m*) that we choose for each of the two subnetworks *NN_sums_* and *NN_prods_*, as described in **Supp. Methods 1.1**. We chose m = 120 for the two models, which resulted in 1708271 total parameters in each model. The relationship between *m*, *n* and the total number of parameters is made more explicit in **Supp. Methods 1.1**. The choice of *m* is determined based on two considertions.

1. We surmised that *m* should roughly scale with *n*, the number of genes being modelled.
2. Given a value of *n*, we selected a plausible range of values for *m*, and picked *m* based on the predictive performance on the validation set. This allows for a means to balance between fitting accuracy and risk of overfitting.

As depicted in **Figure 1** of the main paper, the risk of overfitting is further mitigated by regularization depending on prior knowledge in the modified loss function. The prior knowledge is fed into the model as a network of putative regulator-gene interactions. That is what gives the model a preliminary idea of which regulators should act on a given gene. The model then uses this information to align with the data, while also discovering new regulator-gene interactions from the data itself and removing some connections in the prior.

**Reviewer Comment 3.5** — For the breast cancer data, I am surprised that with one single pseudotime trajectory they can recover such a complicated model. I would like to see more technical details to be convinced that there is no overfitting, and the results are conclusive—that is, one can unambiguously distinguish different competing models.

**Reply**: Note that for training an ODE, the (technical) number of samples are a function of the number of time points rather than the number of patients or replicates as they are trained using pairs of data points. Thus, it is actually not too surprising that training works for a single trajectory. There are, however, two subtle details that we considered in response to the reviewers’ feedback: The high *R*^2^ value was influenced by a large number of constant (flat) trajectories which were easy to predict and, hence, inflated the performance, which was further amplified by an unfortunate choice of testing scheme (see Reviewer Comment 2.10). We addressed both of these issues in the updated manuscript. In response to Reviewer Comment 1.4 we now also provide performance of other methods for the BRCA data, which achieve a similar mean squared error as PHOENIX, but perform much worse in terms of the recovered biologically meaningful signal (see **Figure 6**).

**Reviewer Comment 3.6** — A few comments on the methods they tested:

(a) PRESCIENT uses time courses of snapshot distributions, and assume that one can not fol- low individual cells over time. PRESCIENT assumes a scalar potential governs the systems dynamics. This is generally a questionable assumption for thermodynamic nonequilibrium system (as a cell is), especially when cell cycle is explicitly considered.

I read the preprint of RNAForecaster [(link).](https://www.biorxiv.org/content/10.1101/2022.08.04.502825v2.full) It seems that these authors are also confused about the metabolic labeling method, and assumed that one can measure the mRNA counts of the SAME cell at two time points (Fig. 1 in that preprint). This is incorrect. The ex- perimental methods such as scEU-seq are destructive, so one CANNOT track one cell at two different time points.

**Reply**: Thank you for sharing your thoughts on this matter. We agree that both PRESCIENT and RNAForecaster have potentially questionable assumptions. We hope that our method fills this gap in the context of learning dynamics for longitudinal expression data.

(b) Dynamo: The authors use the average Jacobian to extract a GRN. While this is a method used in the original Dynamo paper, there is a caveat with this method. Notice that for a sigmoidal shaped dose-response function with high nonlinearity (a large Hill coefficient), the

Jacobian is close to zero for both low and high regulator levels—-in the case with a very large Hill coefficient, the absolute value of a Jacobian is significantly larger than 0 only within a small range centered around *K_M_* ; the average Jacobian can be very small if this transition region is not well sampled. The summary in Table 1 is inaccurate.

**Reply**: Thank you for this useful insight. We agree with the point about sampling the Jacobian carefully. To that end we have updated **Supp. Methods 3.3.2** to described this caveat. We also detailed in **Supp. Methods 3.3.2** a modified algorithm for extracting a GRN from Dynamo:

- Instead of sampling random expression vectors ***g****_k_* from the uniform normal distribution

*U* (0*,* 1), we now sample them from *N* (0*.*5*,* 0*.*25). Since high and low expression values corresponded to 1 and 0 in our simulations, sampling from this Gaussian centered around

0.5 made it less likely that we would be picking very high or low regulator levels.

- To further prevent the average Jacobian from being too small, we do sampling in two phases:
  1. We first sample 5000 random expression vectors *{****g****_k_ ∈* R*^n^}*^5000^ from *N* (0*.*5*,* 0*.*25), and calculate the 5000 corresponding Jacobian matrices *{****J****_k_ ∈* R*^n×n^}*^5000^ using dynamo’s get_Jacobian() function. We compute *{|****J****_k_|_F_ ∈* R*}*^5000^, the Frobenius norms for all the Jacobians. Then we chose *f*_cutoff_ = 95^th^ percentile across the 5000 values in

*k*=1

*k*=1

*k*=1

*{|****J****_k_|_F_ }*^5000^ as a filtration criteria for the next step.

*k*=1

2. We sample a **new batch** of 10000 random expression vectors *{****g****_l_}*^10000^ from *N* (0*.*5*,* 0*.*25)

*l*=1

and compute *{****J****_l_}*^10000^. We **discard** any ***J****_l_* that is “not large enough,” that is if

*l*=1

*|****J****_l_|_F_ < f*_cutoff_, and keep sampling until we have 10000 ***J****_l_*s that satisfy this criteria.

- We use the final list of 10000 Jacobian matrices to compute an average Jacobian ***E*** .

We observed that this modified algorithm does indeed marginally improve the AUC for Dynamo (in **Supp. Table 4**), especially in SIM690 where we have a larger number of genes.

(c) Dynamo is not restricted to metabolic labeling data, fewer than 300 genes, and top 30 PC/2D UMAP—the authors might get this wrong impression from some specific examples in that paper. **Reply**: We indeed got this impression from the settings used in the original manuscript.

We now adapted the wording in our manuscript and—also in response to other questions— redesigned original Table 1 (now in Supplement) to reflect that Dynamo as well as other methods are applicable to more genes and without dimensionality reduction, which has just not been demonstrated so far. Furthermore, we added comparisons to competitors on real world data, which also confirms that Dynamo is applicable to more genes and without metabolic labeling.

**Second round of review**

**Reviewer 1**

All my questions have been addressed

**Reviewer 2**

The authors have addressed most of my concerns satisfactorily. I have one minor follow-up request, and am restating one request that I described as essential during the first round of review which I think is not optional and must be performed.

1. To be honest, the newly added supplemental figure 5 is concerning to me. The results from these genes without parameter v indicate that the model is not learning despite large loss gradients. I would believe that this parameter could help stabilize a model that was having difficulty converging around zero gene velocity, but that is evidently not the problem. That there is some arbitrary inability to learn a specific range of values suggests to me a flaw in model design (possible) or training (probable).

However, I do not think that it is appropriate to demand a resolution to this issue at this time, and supplemental figure 5 does clearly report the issue. Please describe the initialization of v in the methods section, and please report for the real-world models in figures 4 and figures 5, the number of genes for which v is <= 0 (and are therefore entirely null in the model).

2. This is a restatement of reviewer point 2.4, which was not adequately addressed. The authors have not shown that this method can recover network edges which are not in the prior P*, only that it can recover some prior edges P* even if other edges are incorrect. The yeast prior P* (motifs) is derived directly from the validation data (ChIP) and does not serve as independent validation.

It is essential that PHOENIX is tested on GRN information that is withheld from the prior P*. The authors must select a set of edges to withhold, remove them from the prior P*, train their model, process it into a GRN, and then report network-level performance only on validation edges that correspond to those edges withheld from P*, which the model is explicitly naive to. It is only necessary to run this control on the simulated tests, and it can be reported as a supplemental figure. It is an important control.

**Authors’ response to reviewers**

# Responses to second-round comments from Reviewer 2

**Reviewer Comment (second-round) 2.1** — To be honest, the newly added **Supp. Fig. 5** is concerning to me. The results from these genes without parameter ***υ*** indicate that the model is not learning despite large loss gradients. I would believe that this parameter could help stabilize a model that was having difficulty converging around zero gene velocity, but that is evidently not the problem. That there is some arbitrary inability to learn a specific range of values suggests to me a flaw in model design (possible) or training (probable). However, I do not think that it is appropriate to demand a resolution to this issue at this time, and **Supp. Fig. 5** does clearly

report the issue. Please describe the initialization of ***υ*** in the methods section, and please report for the real-world models in **Figure 4** and **Figure 5**, the number of genes for which *υ_i_ ≤* 0 (and are therefore entirely null in the model).

**Reply**: This was a thought-provoking set of observations, and we are happy to have the opportunity to provide deeper insight into the model. First, we would like to note that the parameter *υ_i_* does not change the actual model architecture or the functions it learns from the data. Instead, *υ_i_* is an auxiliary variable that influences the training dynamics and enables the NeuralODE model to learn zero gradients more efficiently. Indeed, after training, it can be integrated into the other model parameters without changing the overall learned function.

The *υ_i_*s are initialized as a collection of independent and identically distributed standard uniform values and we have included this information in the section titled **Model formulation and neural network architecture** in the main text. We have also included **Supp. Table 9**, **Supp. Table 12**, and **Supp.**

**Table 16** in which we report the number of genes *i* for which *υ_i_ ≤* 0 in each yeast, breast cancer, and b-

cell model, respectively, that we have inferred using PHOENIX and have referenced these supplementary

tables in the updated captions of **Figure 4** and **Figure 5**.

**Reviewer Comment (second-round) 2.2** — The authors have not shown that this method can recover network edges which are not in the prior *P^∗^*, only that it can recover some prior edges *P^∗^* even if other edges are incorrect. The yeast prior *P^∗^* (motifs) is derived directly from the validation data (ChIP) and does not serve as independent validation. It is essential that PHOENIX is tested

on GRN information that is withheld from the prior *P^∗^*. The authors must select a set of edges to withhold, remove them from the prior *P^∗^*, train their model, process it into a GRN, and then report network-level performance only on validation edges that correspond to those edges withheld from *P^∗^*, which the model is explicitly naive to. It is only necessary to run this control on the

simulated tests, and it can be reported as a supplemental figure. It is an important control.

**Reply**: We performed additional analyses to address this question, the results of which are summarized in. **Supp. Table 2** and referenced in the Results section of the main paper.

For this analysis, we rendered the prior model *P^∗^* “incomplete” by removing *w*% *∈ {*5%*,* 10%*,* 25%*,* 50%*,* 75%*,* 100%*}* of edges from *P^∗^*, hence making PHOENIX explicitly naive *a priori* to that subset of edges (see **Supp. Methods 4.2**). We then calculated network level performance (reported as AUC) on **only**

these *w*% withheld edges based on how well the trained PHOENIX model was able to discriminate

them from non-existent edges and to provide a fair basis of comparison between simulations. For com- pleteness, we separately report AUC values for edges that were **not** withheld from the prior network

*P^∗^*. In general, PHOENIX demonstrates reasonably high performance in recovering the withheld edges (AUC *≥* 0*.*86) while remaining robustly explainable (AUC *≥* 0*.*97) in terms of the edges that were not

withheld. We believe this highlights PHOENIX’s ability to use the prior as a foundation and then extend beyond it to discover new interactions that the prior is explicitly naive to.

We recognize that we could have systematically deleted single edges from each network and then seen how well we recover those edges but felt that this “bulk” edge approach better reflects the likely situation the algorithm would face in analyzing data–one in which there were unknown transcription factor-gene interactions or those that were introduced through genetic variation.

**Third round of review**

**Reviewer 2**

The authors have addressed all of my concerns; I think that this is an excellent method and a well-constructed manuscript and I am happy to endorse it for publication
